# Temporal stability of polymorphic Arctic charr parasite communities reflects sustained divergent trophic niches

**DOI:** 10.1002/ece3.9460

**Published:** 2022-11-05

**Authors:** Eloïse C. Rochat, Rachel A. Paterson, Isabel Blasco‐Costa, Michael Power, Colin E. Adams, Ron Greer, Rune Knudsen

**Affiliations:** ^1^ Department of Arctic and Marine Biology UiT The Arctic University of Norway Tromsø Norway; ^2^ Natural History Museum of Geneva Geneva Switzerland; ^3^ The Norwegian Institute for Nature Research Trondheim Norway; ^4^ Department of Biology University of Waterloo Waterloo Canada; ^5^ Scottish Centre for Ecology & the Natural Environment University of Glasgow Glasgow UK; ^6^ Old Armoury Blair Atholl UK

**Keywords:** introduced species, *Salvelinus alpinus*, Scotland, stable isotopes, trophically transmitted parasites

## Abstract

Polymorphic Arctic charr *Salvelinus alpinus* populations frequently display distinct differences in habitat use, diet, and parasite communities. Changes to the relative species densities and composition of the wider fish community have the potential to alter the habitat niche of sympatric Arctic charr populations. This study evaluated the temporal stability of the parasite community, diet, and stable isotopes (δ^13^C, δ^15^N) of three sympatric Arctic charr morphs (piscivore, benthivore, and planktivore) from Loch Rannoch, Scotland, in relation to changes to the fish community. All Arctic charr morphs displayed distinct differences in parasite communities, diet, and stable isotope signatures over time, despite the establishment of four new trophically transmitted parasite taxa, and increased fish and zooplankton consumption by the piscivorous and planktivore morphs, respectively. Native parasite prevalence also increased in all Arctic charr morphs. Overall, Loch Rannoch polymorphic Arctic charr morph populations have maintained their distinct trophic niches and parasite communities through time despite changes in the fish community. This result indicates that re‐stocking a native fish species has the potential to induce shifts in the parasite community and diet of Arctic charr morphs.

## Graphical abstract

Our study showed that anthropogenic modification of the fish community can result in changes to the parasite fauna in the system, with the establishment of new species and varying levels of infection of native parasites over time. However, our model hosts were able to maintain their trimorphic population despite changes in the fish community.

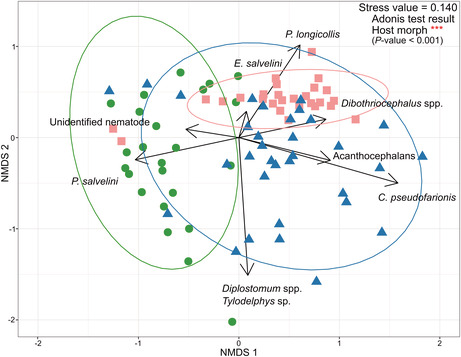

## 1 INTRODUCTION

Freshwater systems are the most threatened ecosystems on earth (Albert et al., 2021; Kernan et al., 2011; Reid et al., 2019; Woodward et al., 2010). Furthermore, human‐induced global change has placed severe pressure on aquatic ecosystem structure and function, and poses a considerable threat to biodiversity (e.g., Christensen et al., 2006; Ficke et al., 2007; Jackson et al., 2016) and food webs (Nagelkerken et al., 2020; Petchey et al., 1999). Model predictions suggest the geographic distributions of many fish species, including Arctic charr (*Salvelinus alpinus* L.), will be significantly reduced as a result of climate warming (Chu et al., 2005; Hein et al., 2012; Svenning et al., 2022). Moreover, given the importance of biological interactions as drivers of ecological processes (Blois et al., 2013), freshwater fish communities have the potential to be highly affected by several other commonly conducted anthropogenic activities. For instance, fish re‐stocking and fish introduction can impact the native fish community (Britton et al., 2011) by modifying inter‐ and intra‐specific competition for food, habitat, and niche space between fish species (e.g., Britton et al., 2010; Gregersen et al., 2006; Klemetsen et al., 2003), parasite component communities (i.e., parasites found in a host population, here in a morph; e.g., “spillback” impact on native fishes; Kelly et al., 2009) and changes in predator–prey relationships (L'Abée‐Lund et al., 1992). Arctic charr is the world's northernmost freshwater fish species (Klemetsen et al., 2003) and thus represents an interesting model species to study the influences in multiple anthropogenetic stressors occurring at the southern edge of this species’ geographic distribution.

Arctic charr express high levels of phenotypic plasticity, with up to five different morphs known to co‐occur in a single water body (Doenz et al., 2019; Skúlason et al., 1989), and may also form genetically segregated populations (Moccetti et al., 2019; Præbel et al., 2016; Simonsen et al., 2017; Verspoor et al., 2010). Sympatric Arctic charr morphs can be distinguished by their size, head morphology, and stable isotope tracers (δ^15^N and δ^13^C), the stable isotope value of which depends on foraging habits (Doenz et al., 2019) and trophic niche (e.g., Adams et al., 2003). Commonly, morphs segregate along the benthic‐pelagic resource axes where one morph feeds in the benthic environment (benthivore morph) and one morph relies more heavily on food available in the water column (planktivore morph; Adams, 1998; Skúlason et al., 1989; Walker et al., 1988). A piscivore Arctic charr morph may occur in either the upper water (Adams, 1998; Sandlund et al., 1992) or deep in the profundal zone (e.g., Knudsen, Amundsen, et al., 2016; Knudsen, Gjelland, et al., 2016; Power et al., 2005).

Arctic charr are known to host more than 40 metazoan parasites (Moravec, 2004). The diversity of their parasite community is attributed to their diet since most known parasite taxa of Arctic charr are trophically transmitted via the consumption of intermediate hosts, often invertebrates or small fish (Moravec, 2004). Thus, Arctic charr morphs are exposed to different parasite communities depending on habitat choice, feeding habits, and the presence of the intermediate hosts in their occupied habitat (Frandsen et al., 1989; Jonsson & Jonsson, 2001; Knudsen et al., 1997; Sandlund et al., 1992). In this context, trophically transmitted parasite communities can reveal the temporal stability of the food web (Behnke et al., 2018), since their complex life cycles span multiple trophic levels.

In this study, we evaluated the temporal stability of the parasite infracommunity of a polymorphic Arctic charr population in Loch Rannoch following brown trout *Salmo trutta* (L.) re‐stocking and crucian carp *Carassius carassius* (L.) introduction (see Fraser & Adams, 1997). A risk exists that introduced fish species bring alien generalist parasites that successfully establish in native fish (e.g., Asian fish tapeworms and yellow grub; Dove et al., 1997; Gaglio et al., 2016; Kuchta et al., 2018), although most parasites tend to be specialized in one or few types of host. While brown trout were already part of the native community, trout originating from a different lake/hatchery are potentially exposed to different parasites, and thus may introduce novel parasite taxa to the system (i.e., a translocation impact: Kelly et al., 2009; Peeler et al., 2011). Arctic charr and brown trout share many parasite species that can be translocated along with the host and establish in the local fish community (e.g., Adolfsen et al., 2021; Bristow, 1993; Knudsen et al., 2007). The parasite load in the system can be indirectly amplified (Kelly et al., 2009) or diluted (Goedknegt et al., 2016) as higher salmonid density can act as an enlarged pool of hosts for native parasites. In addition, the brown trout and crucian carp are two benthivore fish and they could compete for common resources with Arctic charr (Eloranta et al., 2013; Langeland et al., 1991). It is also possible that introduced fishes cause indirect changes in the parasite community of Artic charr through modifying predator–prey links that expose hosts to a different range of parasites or by acting as parasite sinks themselves, thus reducing Arctic charr exposure (Poulin & Mouillot, 2003).

Overall, the Arctic charr parasite community in Loch Rannoch might change through time due to the introduced‐relocated benthivore competitor (e.g., brown trout and crucian carp) or other possible explanations (i.e., annual variability). This increase in benthivore fishes might promote the copepods transmitted parasite (Dorucu, 1996; Dorucu, Adams, et al., 1995). However, the parasite community of the three morphs of Arctic charr might still be different if their diet and habitat are stably diverged through time (Dorucu, 1996). Indeed, we expect that Arctic charr morphs maintain trophic niche partitioning. Thus, we hypothesized that: (a) trophic niches of the three Arctic charr morphs will be stable through time (i.e., diet and isotope); thereby (b) the parasite component communities in Arctic charr morphs will remain distinctly different between morphs as all the taxa previously recorded are trophically transmitted. These two hypotheses are assessed in this study using a contemporary snapshot of the trophic information provided by diet analysis and the proxies of longer‐term trophic niche provided by stable isotope analyses (δ^13^C, δ^15^N) and parasite communities.

## 2 MATERIAL AND METHODS

### 2.1 Study area and samples collection

Loch Rannoch is an oligotrophic lake in the Tayside Region, Scottish Highlands (56°41′N; 004°17′W, 17 km^2^, 203 m above sea level, 134 m maximum depth; Bryce et al., 2016). Loch Rannoch's fish community is composed of eight native fish species (Arctic charr, brown trout that is often re‐stocked, pike *Esox lucius* L., perch *Perca fluviatillis* L., minnow *Phoxinus phoxinus* L., three‐spined stickleback *Gasterosteus aculeatus* L., European eel *Anguilla anguilla* L., Atlantic salmon *Salmo salar* L.; Verspoor et al., 2010; Walker et al., 1988) and an alien species recorded for the first time in 1997, the crucian carp (Fraser & Adams, 1997). Moreover, the Arctic charr population in Loch Rannoch comprises three morphs (a littoral benthivore, planktivore, and profundal piscivore morph; Adams et al. (1998)), which differ in terms of their functional trophic morphologies (Adams & Huntingford, 2002; Bryce et al., 2016), life‐history traits (Adams & Huntingford, 2004; Fraser et al., 2008), trophic niches (Adams et al., 1998) and parasites (Dorucu, 1996). However, the relative amount of each species is unknown.

Arctic charr were sampled using gill nets in October 1992 and July 1993 (*n* = 253; see Dorucu, Adams, et al. (1995), Dorucu (1996) for details), and in October 2010 (this study, *n* = 101). Gill nets were deployed overnight for a maximum period of 12 h during both study periods. Between 1992 and 93, 30 benthivore, 173 planktivore, and 50 piscivore Arctic charr were collected. In 2010, 34 benthivore, 34 planktivore and 33 piscivore Arctic charr were sampled (Table 1). All fish were frozen and transported to the Scottish Centre for Ecology and the Natural Environment (SCENE), University of Glasgow for subsequent analysis (Adams et al., 1998). Fork length (mm) was measured for all sampled fish.

**TABLE 1:** Arctic charr sampled in Loch Rannoch (1992–93 from Dorucu, Adams, et al. (1995) and 2010).

Years	1992–93		2010	
Morph	*N*	Size (min.–max.) mm	*N*	Size (min.–max.) mm
Benthivore	30	194.0 (148–235)	34	206.3 (130–309)
Planktivore	173	186.4 (80–225)	34	234.1 (192–263)
Piscivore	50	191.6 (60–265)	33	288.2 (164–373)

### 2.2 Diet analyses

The stomach fullness was determined from the dissection of the alimentary canal. Stomach contents collected from the upper end of the esophagus to the pyloric sphincter were identified to the lowest practical taxonomic level (typically order or family) under a stereo‐microscope. The diet groups identified in 1992–93 (Dorucu, 1996) served as a reference point for the analyses in 2010 (zooplankton, copepods, surface insects, chironomid larvae, *Pisidium*, insect larvae, *Gammarus*, unidentified invertebrates, fish). The frequency of occurrence of each prey category was evaluated as volume percentage for each stomach and each food category (Hyslop, 1980). Schoener's index (Wallace Jr, 1981) was used as a proxy for diet overlap between different morphs in each time period and the two sampling periods for each morph. This index is usually considered as biologically meaningful when its value exceeds 60% (Wallace Jr, 1981). For comparison, the frequency of occurrences of each prey in 1992–93 was extracted from Dorucu (1996) using DataThief III software (Tummers, 2006).

### 2.3 Parasitological analyses

Parasite prevalence (i.e., proportion of host individuals of an Arctic charr morph that were infected) and mean abundance (i.e., the mean number of parasites in a given host morph) were calculated for each parasite species (Bush et al., 1997). In our study, the parasite prevalence data for Arctic charr from 1992–93 were obtained from Dorucu (1996) using DataThief III software (Tummers, 2006). The fish sampled in 2010 were examined for metazoan parasites using a stereomicroscope, with parasites morphologically identified to species or genera using taxonomical criteria (e.g., Moravec, 2004) before specimens were fixed in absolute ethanol for molecular analyses. We selected some of the specimens used for the morphological analyses and rehydrated them, as preservation in absolute ethanol shrink and/or modify the internal structures of the worms. We prepared whole mounts according to Cribb and Bray (2010) and Justine et al. (2012) protocols. Unfortunately, the preservation quality of the specimens (e.g., poor quality tubes and wrong concentration of alcohol) did not allow a more accurate morphological identification, and molecular identification was needed.

Molecular data were obtained from a subsample of specimens for each prospective parasite taxa (from 2010 only) to confirm their morphological identification. DNA was extracted using Chelex® in deionized water containing 0.1 mg/ml proteinase K. A partial fragment of the large ribosomal subunit (28S rDNA) was chosen as a marker because it is broadly used to molecularly assign parasitic flatworms and acanthocephalans to known genera/species (Blasco‐Costa et al., 2016) and a partial fragment of the small ribosomal subunit (18 S rDNA) was amplified for the nematodes since it is the most common marker used for this group (Černotíková et al., 2011). The following primers were used for the amplification of acanthocephalans, U178 (forward; 5′‐GCA CCC GCT GAA YTT AAG‐3′) and L1642R (reverse; 5′‐CCA GCG CCA TCC ATT TTC A‐3′; Lockyer et al., 2003); and of the nematodes, PhilonemaF (forward; 5′‐GCC TAT AAT GGT GAA ACC GCG AAC‐3′) and PhilPCRr0 (reverse; 5′‐CCG TT CAA GCC ACT GC ATT A‐3′; Černotíková et al., 2011). In addition, the cytochrome c oxidase subunit I mitochondrial gene (COI) was also amplified using Plat‐diploCOX1F (forward; 5′‐CGT TTR AAT TAT ACG GAT CC‐3′) and Plat‐diploCOX1R (reverse; 5′‐AGC ATA GTA ATM GCA GCA GC‐3′; Moszczynska et al., 2009). The PCR amplification protocol for the 28 S marker followed Blasco‐Costa et al. (2009), for the 18 S followed Černotíková et al. (2011) and for the COI followed Blasco‐Costa et al. (2014). Purified amplicons were sent to Macrogen Europe (Amsterdam, Netherlands) for sequencing from both strands, with the same PCR primers used for amplification.

Sequences were assembled and inspected for errors using Geneious® ver. 8.1.9 (Kearse et al., 2012) and submitted to GenBank® (accession numbers in Table S1). Available sequences for taxa belonging to the same family/genus/species as our presumed taxa were obtained from GenBank® and aligned with our sequences to validate species identification or improve the preliminary identification based on specimen morphology. Following this, alignments were obtained using the default parameters in MAFFT (Katoh et al., 2005) and were trimmed at their extremities.

Parasite phylogenetic reconstructions were carried out using maximum likelihood (ML) and Bayesian inference (BI) criteria. The model of nucleotide evolution GTR (general time‐reversible model) with a gamma distribution using among‐site rate variation (Γ) was applied to all analyses. ML analyses were conducted using RAxML ver. 8 (Stamatakis, 2006). All model parameters and bootstrap nodal support values (1000 repetitions) were estimated in RAxML. BI trees were constructed using MrBayes ver. 3.2.6 (Ronquist et al., 2012), running two independent MCMC runs of four chains for 10 million generations and sampling tree topologies every 1000th generation. Burn‐in periods were automatically set to 25,000 generations. RAxML and MrBayes analyses were carried out for each individual dataset on the public computational resource CIPRES (Miller et al., 2011).

### 2.4 Stable isotope analyses

Dorsal muscle tissue samples from 32 benthivore, 32 planktivore, and 21 piscivore Arctic charr sampled in 2010 were dried at 50°C for 24 h, before being ground to a fine powder with a mortar and pestle, and weighed for analysis (0.3 mg). Carbon (δ^13^C) and nitrogen (δ^15^N) isotopes were then analyzed from individual fish at the University of Waterloo, Canada, using a dual inlet Delta Plus Continuous Flow Stable Isotope Ratio Mass Spectrometer (Thermo Finnigan, Bremen, Germany) connected to a Costech Elemental Analyzer (CNSO 4010, Costech Analytical Technologies Inc., Valencia, USA). Obtained stable isotope ratios were expressed in standard delta notation (‰) relative to the international reference materials of Vienna PeeDee Belemnite for carbon (Craig, 1957) and atmospheric nitrogen (Mariotti, 1983). Data quality control was monitored, and corrections were made using a mix of international and in‐house standards (e.g., cellulose and bovine liver) cross‐calibrated against International Atomic Energy Agency standards for Carbon (CH3, CH6) and nitrogen (N1, N2). No <20% of the samples included in any run consisted of standards and reference materials, with obtained measurements used in data normalization and to ensure measurement precision and accuracy. Associated QC/QA checks indicated an error for reportable data of no more than 0.2‰ and 0.3‰, respectively, for δ^13^C and δ^15^N.

### 2.5 Statistical analyses

All the analyses were computed with the statistical software R version 4.1.0 (www.r‐project.org). Separate generalized linear models (GLM) were used to investigate the influence of fish morph (benthivore, planktivore, piscivore) and fish length on species richness, total parasite taxa abundance, and the abundance of each parasite taxon among the three Arctic charr morphs (2010 only). Models were fitted with appropriate Poisson or quasi‐Poisson distributions, to account for over‐dispersion (see Tables S2 and S3) detected by *AER::dispersiontest* (Kleiber & Zeileis, 2008). Additionally, the correlation of variables was assessed using the function *corPlot::psych* (Revelle, 2011) and *cor.test::stat* (R Core Team, 2021), and interactions between variables were included in the GLM models when taxa were correlated with another variable (i.e., fish length in our model).

Dissimilarity among the parasite infracommunities (i.e., parasite taxa abundance of an individual fish) in 2010 as a function of Arctic charr morph (Krebs, 1999) was assessed with nonmetric multidimensional scaling (NMDS) analyses using the zero‐adjusted, Bray–Curtis dissimilarity measure, which is not affected by the number of null values between samples (Clarke et al., 2006). To account for species absences in some infracommunities (zero‐inflated data) a “dummy species” was added to all communities (see Clarke et al. (2006)). The significance of the predictor variables (i.e., fish morph), was tested with the Adonis function. These analyses were conducted with the package *vegan* (Oksanen, 2020) and *MASS* (Venables & Ripley, 2002).

Multivariate analysis of variance tests (MANOVA) and Wilcoxon tests were used to decide whether the distributions of δ^13^C (reflecting the origin of the carbon in the tissue of the fish) and δ^15^N (reflecting its trophic position) values among the three Arctic charr morphs differed (2010 only). The isotopic niche overlaps were also assessed between Arctic charr morphs using a Bayesian approach derived from Swanson et al. (2015) implemented in the package *nicheROVER* (Lysy et al., 2014). This method provides the 95% probability and credibility interval that one individual from one morph could be found within the niche of another morph.

## 3 RESULTS

### 3.1 Stomach contents

**FIGURE 1:**
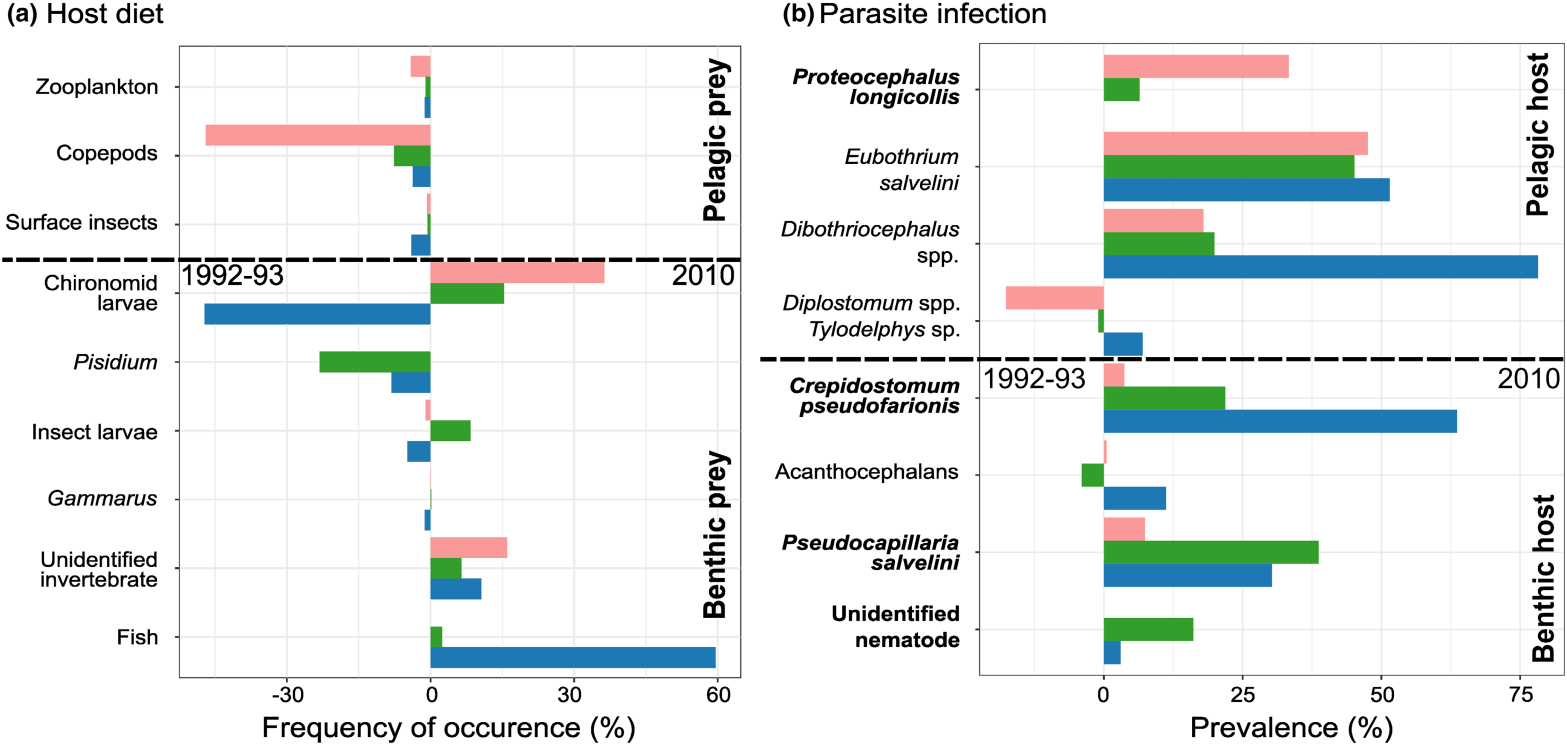
Comparisons between the periods of 1992–93 (at the left) and 2010 (at the right) and among the Arctic charr morphs. (a) Frequency of occurrence of prey items. (b) Parasite infection prevalence. Note that values for the acanthocephalans (*E. truttae* and *N. rutili*)*, Dibothriocephalus* (*D. dendriticus* and *D. ditremus*), and eye flukes (*D. baeri*, *Diplostomum* sp. and *Tylodelphys* sp.) are grouped. Bar colors refer to fish morph: green—benthivore morph; blue—piscivore morph; and pink—planktivore morph.

In 2010, 39.3% of the planktivore morph Arctic charr had empty stomachs, with the remaining individuals having low stomach fullness (mean ± SE; 17.7 ± 5.2%; Figure 1a). This contrasts with the benthivore morph with only 5.7% of individuals having empty stomachs and a stomach fullness of 54.2 ± 5.5%. By contrast, the piscivore morph showed intermediate values of empty stomachs (22.2%) and stomach fullness (31.1 ± 6.4%). Diet differed among the three morphs. Piscivores had a low diet overlap with both the benthivore morph (18.8%) and the planktivore morph (27.8%) due to a high consumption of fish (69.1%). Diet overlap between the benthivore and planktivore morphs (70%) was high as both morphs preyed heavily on chironomid larvae (55.9 ± 5.3% and 52.3 ± 10.7%, respectively). Zooplankton (*Daphnia* and *Polyphemus*) were mainly found in the diet of the planktivore morph (29.7 ± 11.5%). The benthivore morph consumed more *Pisidium* clams (13.6 ± 2.6%), *Gammarus* (0.7 ± 0.3%), and large insect larvae (10.2 ± 5.1%) than the planktivore morph. The largest dietary changes between 1992–93 and 2010 included increased consumption of fish (+59.57%) and decreased chironomid larvae (−47.2%) by the piscivore morph, and reduced consumption of copepods (−47.0%) and increased chironomid larvae (+36.3%) by the planktivore morph (Figure 1a). All other differences between the two sampling periods were minor (<24%).

### 3.2 Parasite communities

Eleven parasite taxa, of which three allogenic taxa that mature in fish and seven autogenic taxa that mature in terrestrial vertebrates (Esch et al., 1988), were identified from Arctic charr sampled in 2010 (Figure 1 and Table 2). The seven species recorded in 1992–93 included the acanthocephalans *Echinorhynchus truttae* (Schrank, 1788) and *Neoechinorhynchus rutili* (Müller, 1780), the trematodes *Diplostomum* spp., and *Tylodelphys* sp.; the cestodes *Dibothriocephalus* spp. (*D. dendriticus* and *D. ditremus*; formerly *Diphyllobothrium* spp. synonymized in Waeschenbach et al. (2017)) and *Eubothrium salvelini* (Schrank, 1790; Dorucu, 1996; Dorucu, Crompton, et al., 1995). In addition to the species above, four taxa were recorded for the first time in 2010, the trematode *Crepidostomum* sp., the cestode *Proteocephalus longicollis* (Zeder, 1800), and two nematodes, *Pseudocapillaria (Ichthyocapillaria) salvelini* (Polyansky, 1952) and one other species unidentifiable based on morphology due to the preservation quality.

**TABLE 2:** Summary of life cycle, hosts, and infection parameters of parasite taxa from three Arctic charr morphs in Loch Rannoch, Scotland, United Kingdom

	Taxa^a^		Infection location	1st intermediate host	2nd intermediate host	Final host	1992–93						2010					
							Benthivore (*n* = 30)		Planktivore (*n* = 173)		Piscivore (50)		Benthivore (*n* = 34)		Planktivore (*n* = 33)		Piscivore (*n* = 34)	
							Prev	MA	Prev	MA	Prev	MA	Prev	MA	Prev	MA	Prev	MA
Trematoda	*C. pseudofarionis*	AU	Intestine	Gastropod or bivalve	Arthropod	Fish	–	–	–	–	–	–	21.9	0.3	3.7	<0.1	63.6	8.0
	*Diplostomum* spp.^b^	AL	Eyes	Gastropod	Fish	Bird	42.2	–	43.0	–	23.9	–	41.2	2.4	6.3	0.1	50.0	6.7
	*Tylodelphys* sp.^b^	AL	Eyes	Gastropod	Fish	Bird												
Cestoda	*P. longicolis*	AU	Intestine	Copepod	None	Fish	–	–	–	–	–	–	6.5	0.1	33.3	1.9	0	–
	*E. salvelini*	AU	Intestine	Copepod	None	Fish	0	–	19.1	–	0	–	45.2	0.6	66.7	1.1	51.5	0.9
	*D. dendriticus*	AL	Stomach, Intestine	Copepod	Fish	Birds, mammals	3.6	–	75.8	–	10.0	–	23.5	0.5	93.8	10.4	88.2	9.3
Acanthocephala	*E. truttae*	AU	Intestine	Amphipod	None	Fish	4.0	–	3.2	–	10.0	–	0	–	3.7	<0.1	21.2	0.3
	*N. rutili*	AU	Intestine	Amphipod	None	Fish												
Nematoda	*P. salvelini*	AU	Stomach, Intestine	Unknown	Oligochaete	Fish	–	–	–	–	–	–	38.7	0.5	7.4	0.1	30.3	0.3
	Unknown nematode	AU	Intestine	Unknown	Unknown	Fish	–	–	–	–	–	–	16.1	0.2	0	–	3.0	<0.1
Taxa richness *S* ^a^							3		4		3		7		7		7	

Abbreviations: AU, autogenic; AL, allogenic; Prev, prevalence %; MA, mean abundance.

^a^ The taxa distinguishable only using a microscope or molecular data were analyzed together: *Diplostomum* spp. with *Tylodelphys* sp.; *E. truttae* with *N. rutili*, and the two *Dibothriocephalus* (*D. dendriticus* with *D. ditremus*).

^b^ Prevalence and abundance estimated from single eye.

**FIGURE 2:**
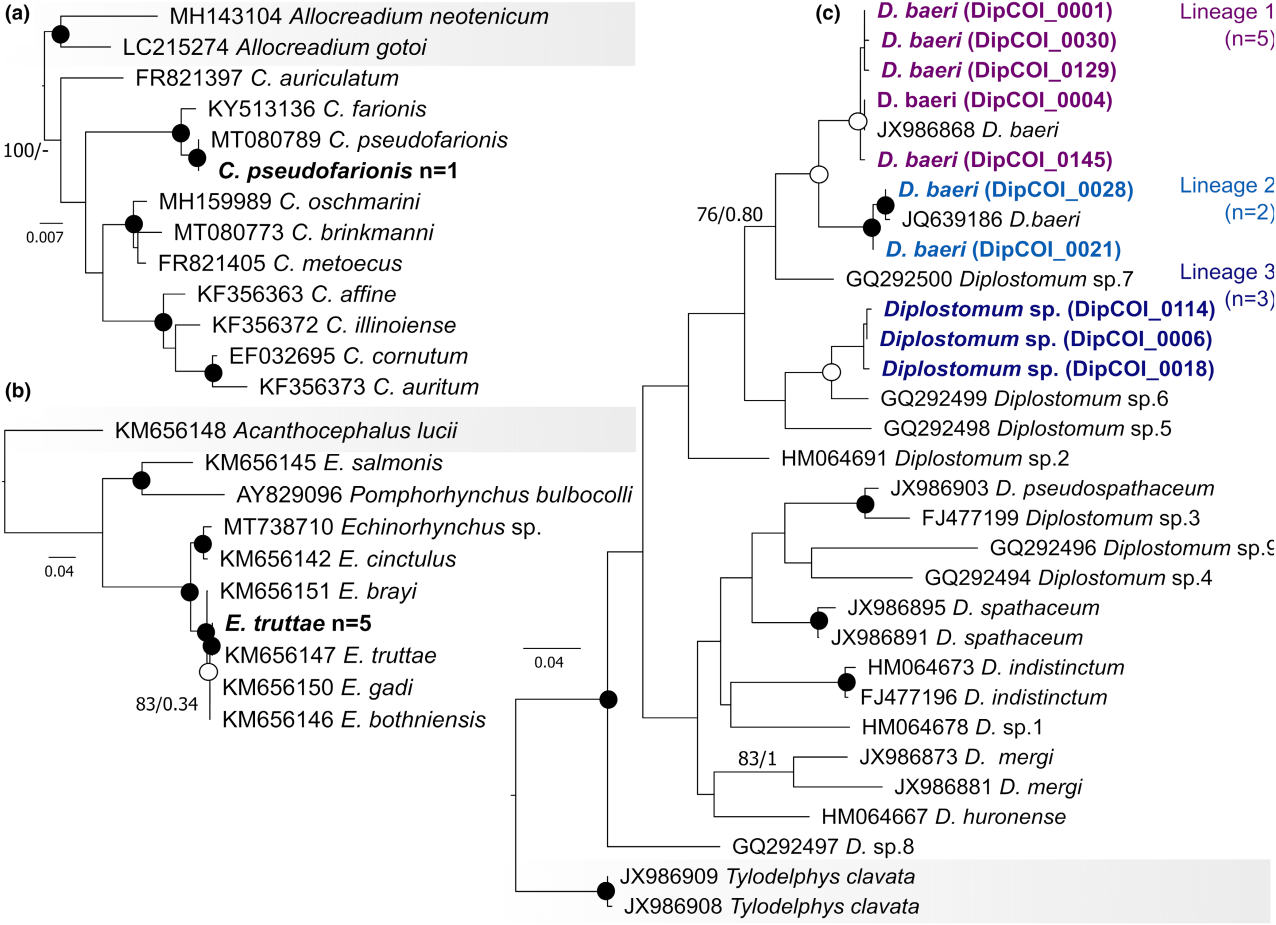
ML phylograms based on partial 28S rRNA or COI mtDNA (c) gene sequences of parasite specimens from this study and selected sequences from GenBank: (a) *Crepidostomum*, with three sequences of taxa belonging to *Allocreadium* as outgroup; (b) *Echinorhynchus*, with a sequence of *Acanthocephalus lucii* as outgroup; (c) *Diplostomum*, with two sequences of *Tylodelphys* as outgroup. Bootstrap values are followed by Bayesian posterior probabilities above the branches. Full circles at the nodes illustrate high support (ML > 90, BI = 1), and empty circles illustrate moderate support (ML = 70–90, BI = 0.90–0.99). Scale bars indicate the number of substitutions per site. Newly acquired sequences are marked in bold, and “*n*” indicates number of specimens sequenced.

Molecular data and phylogenetic analyses corroborated the morphological identification of the new trematode as *Crepidostomum* and further confirmed the identification of the species as *Crepidostomum pseudofarionis* Faltýnková, Pantoja, Skírnisson and Kudlai, 2020 (Figure 2a and GeneBank number: OP580487). The morphological identification of *E. truttae* was also confirmed by molecular methods (Figure 2b and GeneBank number: OP580482 to OP580486). Additionally, three different lineages of *Diplostomum* were molecularly characterized, two of *Diplostomum baeri* Dubois, 1937 and one unidentified *Diplostomum* sp. (Figure 2c and GeneBank number: OP577853 to OP577862). The preservation quality of the specimens of an unknown nematode and *Tylodelphys* sp. did not permit identification to the species level.

### 3.3 Parasite component communities among Arctic charr morphs

In addition to the presence of four previously undetected parasite taxa in Arctic charr, the prevalence of parasites increased in each morph between 1992–93 and 2010 (see Figure 1b and Table 2).

The total prevalence of parasite infection was high in the fish sampled in 2010, 100% of the piscivore and planktivore morphs and 88.2% of the benthivore morph. The piscivore morph showed a significantly higher overall parasite abundance than the benthivore morph, which also showed a higher overall parasite abundance than the planktivore morph (Table 2). The three morphs showed distinctive patterns of infection with differences in parasite prevalence (Figure 1b and Table 2). The eye flukes (*Diplostomum* spp. and *Tylodelphys* sp.) and the two nematode taxa (*P. salvelini* and the unknown nematode) were more common in benthivore morph (see Table 2). The cestode taxa, *Dibothriocephalus* spp. and *E. salvelini* were more common in the planktivores (94 and 68%, respectively) than in the piscivores (88 and 51%) or benthivores (21 and 45%, respectively). *Proteocephalus longicollis* was more common in the planktivores (36%) than in the other morphs. The three remaining taxa (*C. pseudofarionis*, *E. truttae*, and *N. rutili*) were mainly recovered from piscivorous Arctic charr (63% versus <23% in two other morphs for *C. pseudofarionis* and 21% versus <4% in the planktivores for the acanthocephalans). Finally, the two acanthocephalans and the unknown nematode were relatively uncommon (prevalence 0%–21%) in all morphs. Moreover, our results also showed that the overall parasite species richness did not vary between the morphs, but the total abundance of parasites increased with fish length (Table S2).

### 3.4 Parasite infracommunities

**FIGURE 3:**
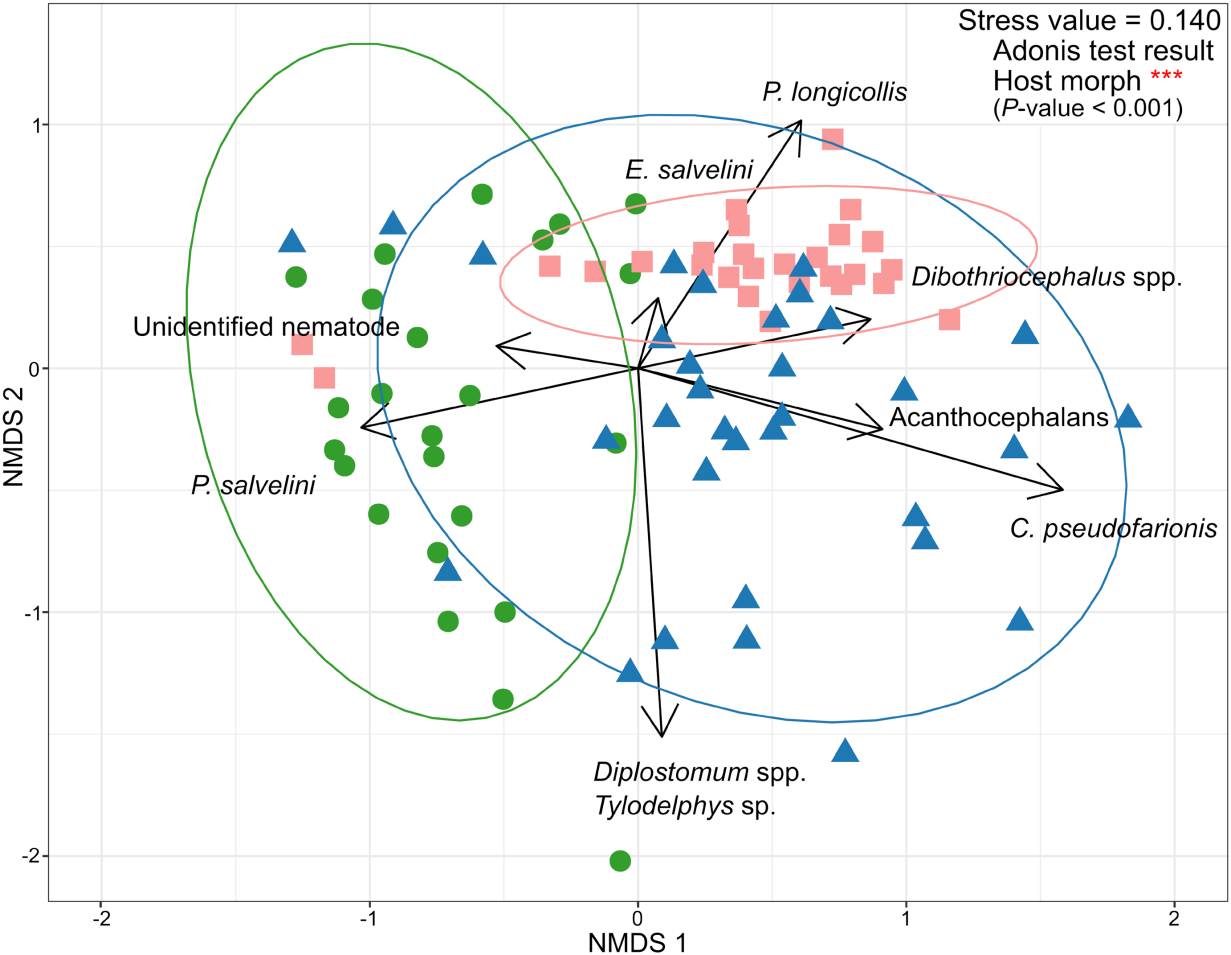
Nonmetrical multidimensional scaling biplot based on Bray–Curtis dissimilarity among parasite infracommunities (*N* = 91). Infracommunities are color‐coded according to the host morph (green—benthivore morph; blue—piscivore morph; pink—planktivore morph). Ellipses regrouped 95% the parasite infracommunities of a particular host morph (colored accordingly). The vectors with arrows in black indicate the contribution of each parasite taxa to the dissimilarity. Random jitter (0.1) was added to the plot to improve visualization of overlapping data points. Asterisks represent *p*‐values lower than .001 for the Adonis test results.

A clear segregation between the parasite communities of the three Arctic charr morphs was found, despite some overlap between the planktivore and piscivore morphs (nonmetric multidimensional scaling analysis; Figure 3). The analysis had a stress value of 0.14, which fell within the accepted range (<0.2; Clarke et al., 2006). Additionally, these were supported by a significant difference (Adonis test; *p*‐value = .001) in the parasite species composition and abundance in the infracommunities between the three morphs. The numerical vectors in the plot show that parasite infracommunities of the benthivore morph were mainly composed of nematodes (*P. salvelini* and an unknown nematode) and the GLM also showed a higher abundance of *P. salvelini* in this morph in contrast with the two other morphs, especially when compared to the planktivores that show the lowest abundance (*t* value = −2.6 and *p*‐value = .011; Table S3). Parasite infracommunities of the planktivore morph were dominated by cestodes (*Dibothriocephalus* spp., *E. salvelini*, and *P. longicollis*; see Table 2) with a much higher abundance of *P. longicollis* than the other two morphs (*t* value = 4.7 and *p*‐value <.001; Table S3). Piscivore‐morph parasite infracommunities were driven by trematodes (*C. pseudofarionis*, *Diplostomum* spp. and *Tylodelphys* sp.; see Table 2) and acanthocephalans (*E. truttae* and *N. rutili*). Moreover, the abundance of autogenic parasite taxa (*Dibothriocephalus* spp., *Diplostomum* spp. and *Tylodelphys* sp.) was significantly linked with fish length (*Dibothriocephalus* spp. in piscivore: *t* value = 5.0 and *p*‐value <.001; eye flukes in benthivore: *t* value = 2.6 and *p*‐value = .011, and piscivore: *t* value = 2.3 and *p*‐value = .021; Table S3).

### 3.5 Stable isotope analysis

**FIGURE 4:**
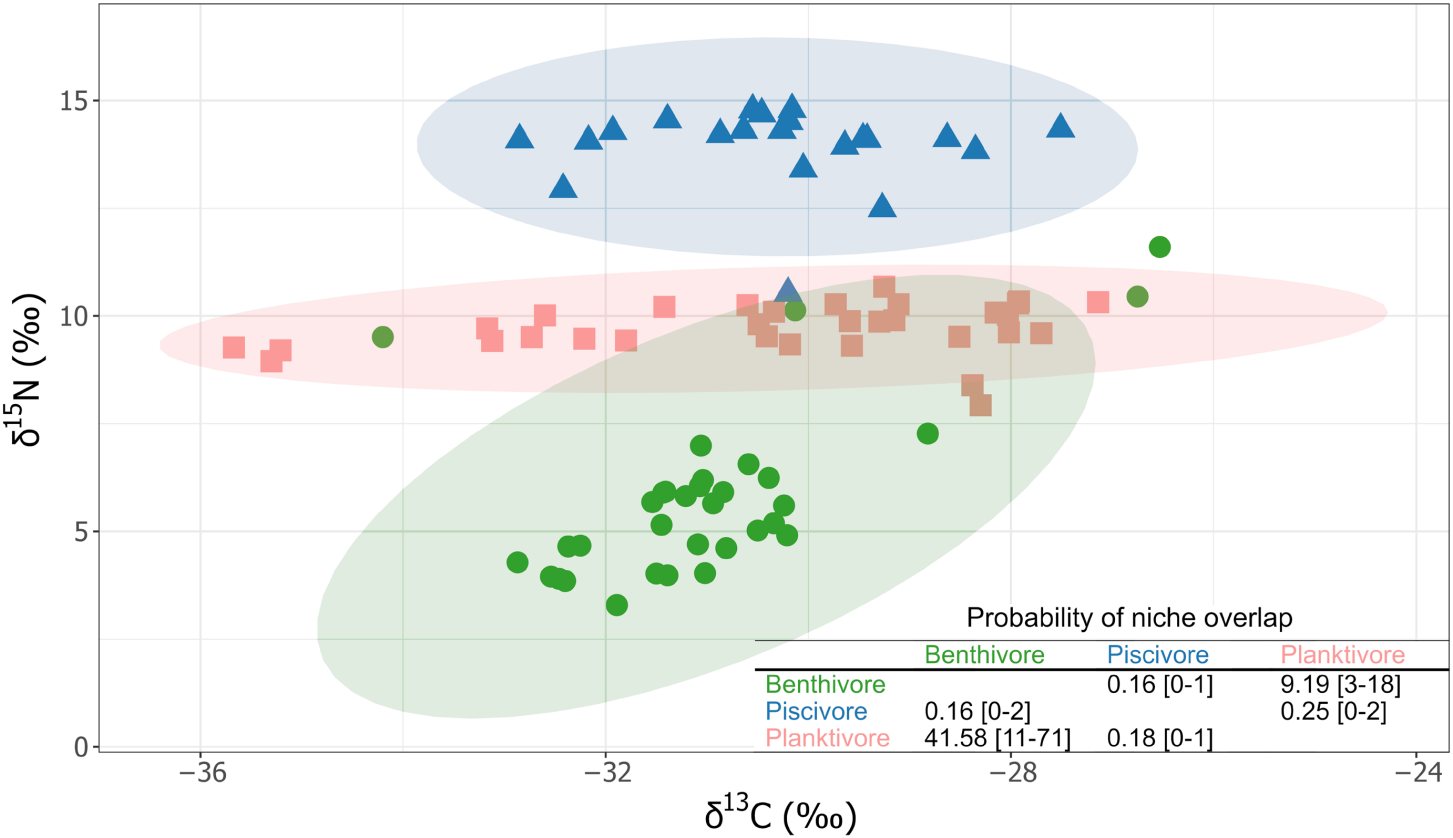
Plot of the stable isotopes δ^13^C and δ^15^N and probabilistic niche overlap calculated using the 95% niche regions between each pair of Arctic charr morphs from Loch Rannoch (2010). Colors refer to fish morph: green—benthivore morph; blue—piscivore morph; and pink—planktivore morph. Ellipses regrouped 95% of the fish of a particular morph (colored accordingly). Mean probability (%) (range 95% credibility interval) of finding an individual of the morph in the row within the niche region of the morph in the column.

Our study was able to evaluate the temporal stability of polymorphic Arctic charr population, despite the limitation of our data, such as the absence of stable isotope data in 1992–93 and the smaller sampling effort in 2010. The stable isotope values showed clear separation of the three Arctic charr morphs mostly based on the δ^15^N values (Figure 4; MANOVA: *F*‐value = 225.23, *p*‐value <.001) rather than the carbon signal. Indeed, there was no significant difference between the three morphs in δ^13^C (MANOVA: *F*‐value = 1.39, *p*‐value = .255). Consistent with its diet, the piscivore morph displayed higher δ^15^N (mean of 13.9 ± 1.0*‰*) values than the plankivore morph (mean of 9.7 ± 0.6*‰*), and the benthivore morph had the lowest δ^15^N values (mean of 5.8 ± 2.0*‰*; Wilcoxon test: *W*‐values = 671 and 1, *p*‐values <.001; Figure 4).

The analyses of the isotopic niche overlap with *α* = 0.95 suggested that the planktivore and benthivore morphs had the highest probabilities of sharing the same trophic region. The mean probability of an individual from the planktivore morph being found in the niche of the benthivore morph was 42 [11–71]% while there was a mean probability of 9 [3–18]% of finding a benthivore morph within the planktivore niche; Figure 4. The probability of either the benthivore or the planktivore morph trophically overlapping with the piscivore morph was low (<1 [0–1]% irrespective of the comparison base, e.g., Figure 4).

## 4 DISCUSSION

Loch Rannoch Arctic charr morphs displayed high trophic niche stability over time. This is reflected in the consistent divergence in the parasite infracommunities and component communities' structure, diet composition, and stable isotope analyses of the three sympatric morphs. Overall, an increase in the prevalence (%) of all native parasite taxa in all three sympatric Arctic charr morphs was recorded between 1992–93 and 2010. Four novel macroparasite taxa were found in 2010, *C. pseudofarionis*, *P. longicollis*, *P. salvelini*, and an unidentified nematode. These parasites are generalist species commonly found as adults in salmonids, (e.g., Chubb, 1963; Dorucu, Crompton, et al., 1995; Moravec, 2004), thus they could have been introduced by the re‐stocking of brown trout. The introduction of crucian carp may not be linked to the presence of these new taxa, as the only parasites are known to infect both crucian carp and Arctic charr are *Diplostomum spathaceum* (Rudolphi, 1819), *Acanthocephalus lucii* (Müller, 1776), and *N. rutili* (Karvonen et al., 2005). Moreover, it is unlikely that these parasite taxa were missed during the first study from Dorucu, Adams, et al. (1995), as the sampling in 1992–93 was larger than in 2010 and the relatively high prevalence of these new parasite taxa.

In our study, the piscivore morph had a low diet overlap with both the benthivore and planktivore morphs, which is consistent with earlier trophic studies in Loch Rannoch (Adams et al., 1998; Dorucu, 1996), and with other studies on similar charr‐morph pairs (Knudsen, Gjelland, et al., 2016; Moccetti et al., 2019). The low diet overlap is supported by the distinct parasite infracommunity composition observed between the two sampling periods (Dorucu, Crompton, et al., 1995), and the markedly different stable isotope values for all morphs from 2010. The planktivore morph had a high diet overlap with the benthivore morph, mainly due to the high consumption of chironomid larvae by both morphs, and the relatively low consumption of zooplankton and copepods by the planktivore morph. The unexpected low zooplankton diet of the planktivore morph contrasted with earlier trophic studies in Loch Rannoch that showed >90% zooplankton in the planktivore diet (Dorucu, 1996; Walker et al., 1988). The difference may relate to the pooling of fish dietary data from October and July in the earlier study (Adams et al., 1998; Dorucu, 1996) and the use of October‐only data in 2010. For example, seasonal abundances of zooplankton in Scottish lochs vary, being higher in June and July than in October (Romo, 1990). However, the high diet overlap between the planktivore and the benthivore morphs did not reflect their long‐term niche divergence, as was indicated by time‐integrated trophic tracers used here in the form of the parasite community and stable isotopes (mainly δ^15^N values) analyses in 2010 that suggest highly diverged dietary niches over a period of several months. Altogether, our results confirm that the trophic niches are divergent between the sympatric morphs and seem to have remained relatively stable through time, i.e., over the 18 years between the two sampling periods used in this study. Similar trophic stability has been reported in other studies of lakes containing two and three sympatric morphs of Arctic charr (e.g., Knudsen et al., 1997; Knudsen et al., 2014; Siwertsson et al., 2016).

The planktivore morph was mainly infected with parasites transmitted by copepods, such as *P. longicollis*, *Dibothriocephalus* spp. and *E. salvelini*, as was noted in the earlier study of these morphs (Dorucu, Adams, et al., 1995). These parasite taxa are typically associated with other planktivore morphs of Arctic charr (Frandsen et al., 1989; Moccetti et al., 2019) and other salmonid species (Chubb, 1982; Knudsen et al., 2003). In addition to possible seasonal differences in availability, the relatively low occurrence of zooplankton prey in the planktivore morph diet could be linked to an introduced competitor of the planktivores such as juveniles crucian carp that feed on plankton (Penttinen & Holopainen, 1992), and juvenile native perch population that have increased in abundance in recent years (C.E. Adams personal com.). Moreover, Loch Rannoch is a relatively small water body and Arctic charr is a highly mobile species. Thus, the possibility of inter‐specific interactions remains despite the potentially low abundance of crucian carp. Additionally, the sampling methods (i.e., time in the gillnets) can impact the occurrence of zooplankton prey in the planktivore morph, as zooplankton digestion will continue as long as the fish is alive in the gillnet. By contrast, infections of *P. longicollis* and *Dibothriocephalus* spp. were low in the benthivore morph (6 and 21%, respectively) suggesting a low consumption of zooplankton by this morph over time. The prevalent infection of *C. pseudofarionis* trematodes (23%) and *P*. *salvelini* nematode (39%) transmitted by insect larvae or amphipods and oligochaetes, respectively, were expected because the benthivore morph diet is apparently dominated by benthic prey.

Benthivore‐specialized morphs of Arctic charr commonly have parasite communities that are clearly divergent from sympatric planktivore morphs (Moccetti et al., 2019; Siwertsson et al., 2016). The parasite community of benthivore morphs is mainly composed of taxa that use benthic species as intermediate hosts, such as snails, insect larvae, and amphipods (Knudsen et al., 2014). The divergence of the δ^15^N stable isotope signals noted in this study corroborates the dissimilar parasite communities and the diet differences of the benthivore and planktivore morphs.

Parasite community studies of reproductively isolated piscivore morphs of Arctic charr are rare (but see Siwertsson et al., 2016; Moccetti et al., 2019). In Loch Rannoch, *E. salvelini* (52%) and *Dibothriocephalus* spp. (88%) show an increase in infection prevalence from 1992–93 to 2010. In agreement with previous studies, the diet of the piscivore morph consists mainly of fish, as supported by an elevated level δ^15^N isotope in comparison to other morphs and the low niche overlap values (e.g., Knudsen, Amundsen, et al., 2016; McCarthy et al., 2004; Power et al., 2005). Additionally, a rather high prevalence of *C. pseudofarionis* (64%), *P. salvelini* (74%), and acanthocephalans (21%), all transmitted via benthic prey consumption, suggests that benthos (amphipods, oligochaetes, insect larvae) are also relatively common food items in piscivores and our finding corroborate previous observations (e.g., Moccetti et al., 2019).

There has been a general increase in infection levels of the native parasite taxa in all the Arctic charr morphs over time. In 2010, the eye flukes (*Diplostomum* spp. and *Tylodelphys* sp.) had the lowest infection in the planktivore morph, probably because the parasite larvae swarm from the *Radix* sp. snail populations in the littoral zone exposing the benthivore and piscivore morphs to highest infection pressures. The acanthocephalans (two species, Dorucu, Crompton, et al. (1995)) were infrequent in both time periods, mirroring a low feeding rate on *Gammarus*, the intermediate hosts for *E. truttae* and *N. rutili*. For the cestodes, *Dibothriocephalus* spp. and *E. salvelini*, prevalence increased distinctly between 1992–93 and 2010 for all morphs (up to 94%). Both these taxa can be transmitted to the fish host either by feeding on copepods directly or through feeding on fish prey (with parasite re‐establishment in the predator). The increase in prevalence suggests altered transmission rates resulting from either change in the zooplankton community and/or the prey fish community. Re‐establishment from prey fish explains the very high infection of these two parasite species in the piscivore morph in 2010, which were feeding intensively on fish.

The newly recorded parasite taxa (i.e., *C. pseudofarionis*, *P. salvelini*, *P. longicollis*, and the unknown nematode) in 2010 should be regarded as commonly occurring (prevalence up to 73%). These parasite taxa are easy to identify due to their distinct shape and size, and were unlikely to have been missed in the earlier 1992–3 study. *Proteocephalus longicollis* and *C. pseudofarionis* are common and relatively abundant in many salmonids, as well as in brown trout and Arctic charr (Moravec, 2004; Scholz & Hanzelova, 1998; Soldánová et al., 2017). Their presence is most likely attributed to the stocking of brown trout in the lake in previous years, with three of the new parasite species (i.e., *C. pseudofarionis*, *P*. *salvelini*, *P. longicollis*) known to infect different brown trout populations in the UK (Hartvigsen & Kennedy, 1993; Kennedy, 1978). None of these potentially introduced parasite species are regarded as problematic for Loch Rannoch Arctic charr. *Crepidostomum pseudofarionis* has not been previously reported as pathogenic for its final fish host (Moravec, 2004) and *P. longicollis* is usually not or only slightly pathogenic (Bauer et al., 1977; Moravec, 2001a; Scholz, 1999). Additionally, although capillariids are generally considered to be pathogenic at high infection levels, *P. salvelini* have not previously been reported as problematic (Moravec, 2001b; Moravec, 2004) and their abundances were generally low even in the most infected Arctic charr morph.

Arctic charr morphs in Loch Rannoch have shown an increase in parasite infections, as native parasite taxa have become more prevalent and new taxa have been established. Previous fish studies have shown relatively stable infection of trophic‐transmitted parasite taxa through time (e.g., Kennedy, 2001; Kuhn et al., 2016). Changed transmission rates may have been caused by alterations in the food‐web structure, for example, copepod transmitted parasites (Henriksen et al., 2019; Lopez & Duffy, 2021) related to human disturbances. In Loch Rannoch, one indirect cause of change could be the introduction of an alien fish species (e.g., crucian carp: Fraser & Adams, 1997) by fisherman and the increase in benthivore competitors (e.g., brown trout and perch), which may have altered the inter‐specific competition between fish species and changed predator–prey relationships (e.g., Britton et al., 2010; Gregersen et al., 2006; Klemetsen et al., 2003), and thereby changed transmission rates and routes of parasites to the three Arctic charr morphs. As the parasite communities of crucian carp are very different from salmonids (Karvonen et al., 2005), it is unlikely that crucian carp and Arctic charr share parasite taxa. Introductions of fish or crustaceans have in many cases been shown to change lake ecosystems in the United Kingdom (Adams, 1994; Adams & Mitchell, 1992) but have also changed interactions between native sympatric species/morphs (Taylor et al., 2006) including Arctic charr morphs (Knudsen et al., 2019). Indeed, if the population of benthivore (e.g., crucian carp, brown trout, and perch) increases in future, it could affect the benthivore and planktivore morphs of Arctic charr. For instance, the introduced crucian carp predominantly feed on chironomid larvae and benthic cladocerans (among other benthic invertebrates) and could potentially compete with Arctic charr (Adams et al., 1998; Fraser & Adams, 1997; Penttinen & Holopainen, 1992). However, the introduction of crucian carp in the system should not be the main vector of the change in the parasite community of Arctic charr as they are not abundant in the lake (C.E. Adams pers.com). Alterations in the native fish community (e.g., trout stocking or increases in the perch population) may also initiate cascades in the food‐web structure and Arctic charr niche (Sandlund et al., 2016). Then, the changes in the fish community might subsequently directly and indirectly change the parasite community structure of native (i.e., Arctic charr) fish hosts (e. g. Amundsen et al., 2013; Kelly et al., 2009; Kuhn et al., 2015). This change in the parasite community has also likely happened in the fish community of Loch Rannoch.

Overall, the Arctic charr populations in Loch Rannoch have lower infection levels of helminths compared with other northern lake systems (Amundsen et al., 2015; Kuhn et al., 2015; Paterson et al., 2018). Unfortunately, the intensity of parasite infections from the earlier study on Loch Rannoch system was not reported by Dorucu, Adams, et al. (1995), thereby preventing an assessment of the evolution of the parasite load through time. However, even if none of the potentially introduced species (i.e., *C. pseudofarionis*, *P. salvelini*, *P. longicollis*) are considered as highly detrimental to Arctic charr, the increase in infection of the native *Dibothriocephalus* spp. and *E. salvelini* may hamper growth and increase the negative impact at the individual or population level (Boyce, 1979; Curtis, 1984; Saksvik et al., 2001). Some piscivore and planktivore morph individuals from Loch Rannoch had *Dibothriocephalus* spp. infection intensities are known to have deleterious effects on Arctic charr elsewhere (e.g., inhibiting gonadal development; Curtis, 1984; Blanar et al., 2005). Given that Loch Rannoch supports the only known population of a piscivore morph of Arctic charr in Scotland (Adams et al., 1998; Gardner et al., 1988; Walker et al., 1988), an increased parasite load along with additional anthropogenic‐induced stressors (e.g., fishing activity) could pose threats to the unique Arctic charr populations supported by this lake (Adams, 1994, 1996; Fraser & Adams, 1997).

In conclusion, the habitat, and the trophic behavior of the Arctic charr morphs in Loch Rannoch seem to be relatively stable through time. The parasite component communities remain distinct among the three Arctic charr morphs regardless of modifications to the fish community. However, the establishment of new parasite taxa (four species) and a general increase in infection load may represent altered negative effects on the local populations of Arctic charr morphs in the future.

## AUTHOR CONTRIBUTIONS

**Eloïse Coralie Rochat:** Formal analysis (lead); funding acquisition (supporting); investigation (lead); methodology (lead); project administration (equal); validation (equal); visualization (equal); writing – original draft (lead); writing – review and editing (lead). **Rachel Anne Paterson:** Formal analysis (equal); funding acquisition (lead); investigation (equal); methodology (equal); supervision (equal); validation (equal); visualization (equal); writing – original draft (equal); writing – review and editing (equal). **Isabel Blasco‐Costa:** Formal analysis (equal); methodology (equal); supervision (equal); validation (equal); writing – original draft (equal); writing – review and editing (equal). **Micheal Power:** Investigation (equal); methodology (equal); writing – original draft (equal); writing – review and editing (equal). **Colin E. Adams:** Conceptualization (equal); data curation (lead); funding acquisition (supporting); project administration (equal); writing – original draft (equal); writing – review and editing (equal). **Ron Greer:** Data curation (lead); writing – review and editing (supporting). **Rune Knudsen:** Conceptualization (lead); data curation (equal); formal analysis (equal); funding acquisition (equal); methodology (equal); supervision (equal); validation (equal); writing – original draft (equal); writing – review and editing (equal).

## FUNDING INFORMATION

The genetic analyses of *Diplostomum* were funded by the Cardiff University School of Biosciences Seedcorn Fund; AquaWales; the European Union's Horizon 2020 Research and Innovation Programme under the Marie Skłodowska‐Curie grant agreement (no. 663830).

## CONFLICT OF INTEREST

None declared.

### OPEN RESEARCH BADGES

This article has earned an Open Data badge for making publicly available the digitally‐shareable data necessary to reproduce the reported results. The data is available at https://doi.org/10.5061/dryad.jdfn2z3f2.

## Supporting information


Table S1.
Table S2.
Table S3.

## Data Availability

Data from the manuscript is publically available in the Dryad database (https://doi.org/10.5061/dryad.jdfn2z3f2). The DNA sequences will be submitted to Genbank (https://www.ncbi.nlm.nih.gov/genbank/).

## References

[ece39460-bib-0001] Adams, C. , Fraser, D. , Huntingford, F. , Greer, R. , Askew, C. , & Walker, A. (1998). Trophic polymorphism amongst Arctic charr from Loch Rannoch, Scotland. Journal of Fish Biology, 52, 1259–1271.

[ece39460-bib-0002] Adams, C. , Fraser, D. , McCarthy, I. , Shields, S. , Waldron, S. , & Alexander, G. (2003). Stable isotope analysis reveals ecological segregation in a bimodal size polymorphism in Arctic charr from Loch Tay, Scotland. Journal of Fish Biology, 62, 474–481.

[ece39460-bib-0003] Adams, C. , & Mitchell, J. (1992). Introduction of another non‐native fish species to Loch Lomond: Crucian carp (*Carassius carassius* (L.)). Glasgow Naturalist, 22, 165–168.

[ece39460-bib-0004] Adams, C. E. (1994). The fish community of Loch Lomond, Scotland: Its history and rapidly changing status. Hydrobiologia, 290, 91–102.

[ece39460-bib-0005] Adams, C. E. (1996). The impact of introductions of new fish species on predator—prey relationships in freshwater lakes. In S. P. R. Greenstreet & M. L. Tasker (Eds.), Aquatic Predators and their Prey (pp. 98–106). Fishing News Books.

[ece39460-bib-0006] Adams, C. E. (1998). Does the underlying nature of polymorphism in the Arctic charr differ across the species. International Society of Arctic Char Fanatics Information Series, 7, 61–67.

[ece39460-bib-0007] Adams, C. E. , & Huntingford, F. A. (2002). The functional significance of inherited differences in feeding morphology in a sympatric polymorphic population of Arctic charr. Evolutionary Ecology, 16, 15–25.

[ece39460-bib-0008] Adams, C. E. , & Huntingford, F. A. (2004). Incipient speciation driven by phenotypic plasticity? Evidence from sympatric populations of Arctic charr. Biological Journal of the Linnean Society, 81, 611–618.

[ece39460-bib-0009] Adolfsen, P. , Bardal, H. , & Aune, S. (2021). Fighting an invasive fish parasite in subarctic Norwegian rivers–The end of a long story. Management of Biological Invasions, 12, 49–65.

[ece39460-bib-0010] Albert, J. S. , Destouni, G. , Duke‐Sylvester, S. M. , Magurran, A. E. , Oberdorff, T. , Reis, R. E. , Winemiller, K. O. , & Ripple, W. J. (2021). Scientists' warning to humanity on the freshwater biodiversity crisis. Ambio, 50, 85–94. 32040746 10.1007/s13280-020-01318-8 PMC7708569

[ece39460-bib-0011] Amundsen, P. A. , Lafferty, K. D. , Knudsen, R. , Primicerio, R. , Kristoffersen, R. , Klemetsen, A. , & Kuris, A. M. (2013). New parasites and predators follow the introduction of two fish species to a subarctic lake: Implications for food‐web structure and functioning. Oecologia, 171, 993–1002. 10.1007/s00442-012-2461-2 23053223 PMC3612402

[ece39460-bib-0012] Amundsen, P.‐A. , Smålås, A. , Kristoffersen, R. , Knudsen, R. , Siwertsson, A. , & Klemetsen, A. (2015). Takvatnprosjektet – Forskning og kultivering av en overbefolka røyebestand. Septentrio Academic Publishing. 10.7557/7.3420.

[ece39460-bib-0013] Bauer, O. , Musselius, V. , Nikolaeva, V. , & Strelkov, Y. A. (1977). Ichthyopathology. Pishchevaya Promyshlenost

[ece39460-bib-0014] Behnke, J. M. , Bajer, A. , Behnke‐Borowczyk, J. , Clisham, N. , Gilbert, F. , Glover, A. , Jeffery, L. , Kirk, J. , Mierzejewska, E. J. , & Mills, S. C. (2018). Long‐term spatiotemporal stability and dynamic changes in helminth infracommunities of spiny mice (*Acomys dimidiatus*) in St. Katherine's Protectorate, Sinai, Egypt. Parasitology, 146, 50–73. 29921333 10.1017/S0031182018000987

[ece39460-bib-0015] Blanar, C. A. , Curtis, M. , & Chan, H. (2005). Growth, nutritional composition, and hematology of Arctic charr (*Salvelinus alpinus*) exposed to toxaphene and tapeworm (*Diphyllobothrium dendriticum*) larvae. Archives of Environmental Contamination and Toxicology, 48, 397–404. 15719195 10.1007/s00244-004-0064-6

[ece39460-bib-0016] Blasco‐Costa, I. , Balbuena, J. A. , Kostadinova, A. , & Olson, P. D. (2009). Interrelationships of the Haploporinae (Digenea: Haploporidae): A molecular test of the taxonomic framework based on morphology. Parasitology International, 58, 263–269. 10.1016/j.parint.2009.03.006 19345743

[ece39460-bib-0017] Blasco‐Costa, I. , Cutmore, S. C. , Miller, T. L. , & Nolan, M. J. (2016). Molecular approaches to trematode systematics: ‘best practice' and implications for future study. Systematic Parasitology, 93, 295–306. 10.1007/s11230-016-9631-2 26898592

[ece39460-bib-0018] Blasco‐Costa, I. , Faltynkova, A. , Georgieva, S. , Skirnisson, K. , Scholz, T. , & Kostadinova, A. (2014). Fish pathogens near the Arctic Circle: Molecular, morphological and ecological evidence for unexpected diversity of *Diplostomum* (Digenea: diplostomidae) in Iceland. International Journal for Parasitology, 44, 703–715. 10.1016/j.ijpara.2014.04.009 24929135

[ece39460-bib-0019] Blois, J. L. , Zarnetske, P. L. , Fitzpatrick, M. C. , & Finnegan, S. (2013). Climate change and the past, present, and future of biotic interactions. Science, 341, 499–504. 23908227 10.1126/science.1237184

[ece39460-bib-0020] Boyce, N. (1979). Effects of *Eubothrium salvelini* (Cestoda: Pseudophyllidea) on the growth and vitality of sockeye salmon, *Oncorhynchus nerka* . Canadian Journal of Zoology, 57, 597–602.

[ece39460-bib-0021] Bristow, G. A. (1993). Parasites of norwagian freshwater salmonids and interactions with farmed salmon – a review. Fisheries Research, 17, 219–227. 10.1016/0165-7836(93)90021-x

[ece39460-bib-0022] Britton, J. R. , Davies, G. D. , & Harrod, C. (2010). Trophic interactions and consequent impacts of the invasive fish *Pseudorasbora parva* in a native aquatic foodweb: A field investigation in the UK. Biological Invasions, 12, 1533–1542.

[ece39460-bib-0023] Britton, R. , Gozlan, R. , & Copp, G. (2011). Managing non‐native fish in the environment. Fish and Fisheries, 12, 256–274. 10.1111/j.1467-2979.2010.00390.x

[ece39460-bib-0024] Bryce, C. , Fraser, A. , Knudsen, R. , Greer, R. , & Adams, C. (2016). Divergent functional traits in three sympatric Arctic charr *Salvelinus alpinus* morphs are not coupled with the age of the lineage divergence. Hydrobiologia, 783, 177–189. 10.1007/s10750-016-2964-7

[ece39460-bib-0025] Bush, A. O. , Lafferty, K. D. , Lotz, J. M. , & Shostak, A. W. (1997). Parasitology meets ecology on its own terms: Margolis *et al*. revisited. Journal of Parasitology, 83, 575–583. 10.2307/3284227 9267395

[ece39460-bib-0026] Černotíková, E. , Horák, A. , & Moravec, F. (2011). Phylogenetic relationships of some spirurine nematodes (Nematoda: Chromadorea: Rhabditida: Spirurina) parasitic in fishes inferred from SSU rRNA gene sequences. Folia Parasitologica, 58, 135–148. 21776893

[ece39460-bib-0027] Christensen, M. R. , Graham, M. D. , Vinebrooke, R. D. , Findlay, D. L. , Paterson, M. J. , & Turner, M. A. (2006). Multiple anthropogenic stressors cause ecological surprises in boreal lakes. Global Change Biology, 12, 2316–2322. 10.1111/j.1365-2486.2006.01257.x

[ece39460-bib-0028] Chu, C. , Mandrak, N. E. , & Minns, C. K. (2005). Potential impacts of climate change on the distributions of several common and rare freshwater fishes in Canada. Diversity and Distributions, 11, 299–310.

[ece39460-bib-0029] Chubb, J. C. (1963). On the characterization of the parasite fauna of the fish of Llyn Tegid. Proceedings of the Zoological Society of London, 141, 609–621.

[ece39460-bib-0030] Chubb, J. C. (1982). Seasonal occurrence of helminths in freshwater fishes Part IV. Adult Cestoda, Nematoda and Acanthocephala. Advances in Parasitology, 20, 1–292. 6765855 10.1016/s0065-308x(08)60539-4

[ece39460-bib-0031] Clarke, K. R. , Somerfield, P. J. , & Chapman, M. G. (2006). On resemblance measures for ecological studies, including taxonomic dissimilarities and a zero‐adjusted Bray–Curtis coefficient for denuded assemblages. Journal of Experimental Marine Biology and Ecology, 330, 55–80. 10.1016/j.jembe.2005.12.017

[ece39460-bib-0032] Craig, H. (1957). Isotopic standards for carbon and oxygen and correction factors for mass‐spectrometric analysis of carbon dioxide. Geochimica et Cosmochimica Acta, 12, 133–149.

[ece39460-bib-0033] Cribb, T. H. , & Bray, R. A. (2010). Gut wash, body soak, blender and heat‐fixation: Approaches to the effective collection, fixation and preservation of trematodes of fishes. Systematic Parasitology, 76, 1–7. 10.1007/s11230-010-9229-z 20401574

[ece39460-bib-0034] Curtis, M. (1984). *Diphyllobothrium* spp. and the Arctic charr: Parasite acquisition and its effects on a lake‐resident population. In L. Johnson & B. Burns (Eds.), Biology of the Arctic charr. Proceedings of the International Symposium on a Arctic charr, Winnipeg, Mannitoba (pp. 395–411). University of Manitoba Press.

[ece39460-bib-0035] Doenz, C. J. , Krähenbühl, A. K. , Walker, J. , Seehausen, O. , & Brodersen, J. (2019). Ecological opportunity shapes a large Arctic charr species radiation. Proceedings of the Royal Society B‐Biological Sciences, 286, 20191992. 10.1098/rspb.2019.1992 PMC6834057 31640512

[ece39460-bib-0036] Dorucu, M. (1996). Ecology of helminth infections in salmonid fish (p. 247). University of Glasgow.

[ece39460-bib-0037] Dorucu, M. , Adams, C. , Huntingford, F. , & Crompton, D. (1995). How fish‐helminth associations arise: An example from Arctic charr in Loch Rannoch. Journal of Fish Biology, 47, 1038–1043.

[ece39460-bib-0038] Dorucu, M. , Crompton, D. W. T. , Huntingford, F. A. , & Walters, D. E. (1995). The ecology of endoparasitic helminth infections of brown trout (*Salmo trutta*) and rainbow trout (*Oncorhynchus mykiss*) in Scotland. Folia Parasitologica, 42, 29–35. 9599425

[ece39460-bib-0039] Dove, A. D. M. , Cribb, T. H. , Mockler, S. P. , & Lintermans, M. (1997). The Asian fish tapeworm, *Bothriocephalus acheilognathi*, in Australian freshwater fishes. Marine and Freshwater Research, 48, 181–183. 10.1071/mf96069

[ece39460-bib-0040] Eloranta, A. P. , Knudsen, R. , & Amundsen, P. A. (2013). Niche segregation of coexisting Arctic charr (*Salvelinus alpinus*) and brown trout (*Salmo trutta*) constrains food web coupling in subarctic lakes. Freshwater Biology, 58, 207–221. 10.1111/fwb.12052

[ece39460-bib-0041] Esch, G. W. , Kennedy, C. R. , Bush, A. O. , & Aho, J. M. (1988). Patterns in helminth communities in fresh–water fish in Great Britain: Alternative strategies for colonization. Parasitology, 96, 519–532. 10.1017/s003118200008015x 3405638

[ece39460-bib-0042] Ficke, A. D. , Myrick, C. A. , & Hansen, L. J. (2007). Potential impacts of global climate change on freshwater fisheries. Reviews in Fish Biology and Fisheries, 17, 581–613. 10.1007/s11160-007-9059-5

[ece39460-bib-0043] Frandsen, F. , Malmquist, H. J. , & Snorrason, S. S. (1989). Ecological parasitology of polymorphic Arctic charr, *Salvelinus alpinus* (L.), in Thingvallavatn, Iceland. Journal of Fish Biology, 34, 281–297.

[ece39460-bib-0044] Fraser, D. , & Adams, C. E. (1997). A crucian carp *Carassius carassius* (L.) in Loch Rannoch, Scotland: Further evidence of the threat posed to unique fish communities by introduction of alien fish species. Aquatic Conservation: Marine and Freshwater Ecosystems, 7, 323–326. 10.1002/(sici)1099-0755(199712)7:4<323::Aid-aqc252>3.0.Co;2-k

[ece39460-bib-0045] Fraser, D. , Huntingford, F. , & Adams, C. (2008). Foraging specialisms, prey size and life‐history patterns: A test of predictions using sympatric polymorphic Arctic charr (*Salvelinus alpinus*). Ecology of Freshwater Fish, 17, 1–9.

[ece39460-bib-0046] Gaglio, G. , Reina, V. , Caffara, M. , Gjurcevic, E. , Iaria, C. , & Marino, F. (2016). Risk of introduction of *Clinostomum complanatum* (Digenea: Clinostomidae) to Sicily through use of *Cobitis bilineata* (Canestrini, 1865) as live baits. Bulletin of the European Association of Fish Pathologists, 36, 105–110.

[ece39460-bib-0047] Gardner, A. , Walker, A. , & Greer, R. (1988). Morphometric analysis of two ecologically distinct forms of Arctic charr, *Salvelinus alpinus* (L.), in Loch Rannoch, Scotland. Journal of Fish Biology, 32, 901–910.

[ece39460-bib-0048] Goedknegt, M. A. , Feis, M. E. , Wegner, K. M. , Luttikhuizen, P. C. , Buschbaum, C. , Camphuysen, K. C. , Van der Meer, J. , & Thieltges, D. W. (2016). Parasites and marine invasions: Ecological and evolutionary perspectives. Journal of Sea Research, 113, 11–27.

[ece39460-bib-0049] Gregersen, F. , Aass, P. , Vøllestad, L. , & L'Abée‐Lund, J. (2006). Long‐term variation in diet of Arctic char, *Salvelinus alpinus*, and brown trout, *Salmo trutta*: Effects of changes in fish density and food availability. Fisheries Management and Ecology, 13, 243–250.

[ece39460-bib-0050] Hartvigsen, R. , & Kennedy, C. R. (1993). Paterns in the composition and richness of helminth communities in brown trout, *Salmo trutta*, in a group of reservoirs. Journal of Fish Biology, 43, 603–615.

[ece39460-bib-0051] Hein, C. L. , Öhlund, G. , & Englund, G. (2012). Future distribution of Arctic char *Salvelinus alpinus* in Sweden under climate change: Effects of temperature, lake size and species interactions. Ambio, 41, 303–312. 22864703 10.1007/s13280-012-0308-z PMC3535054

[ece39460-bib-0052] Henriksen, E. H. , Frainer, A. , Knudsen, R. , Kristoffersen, R. , Kuris, A. M. , Lafferty, K. D. , & Amundsen, P. A. (2019). Fish culling reduces tapeworm burden in Arctic charr by increasing parasite mortality rather than by reducing density‐dependent transmission. Journal of Applied Ecology, 56, 1482–1491. 10.1111/1365-2664.13369

[ece39460-bib-0053] Hyslop, E. J. (1980). Stomach contents analysis – a review of methods and their application. Journal of Fish Biology, 17, 411–429.

[ece39460-bib-0054] Jackson, M. C. , Loewen, C. J. G. , Vinebrooke, R. D. , & Chimimba, C. T. (2016). Net effects of multiple stressors in freshwater ecosystems: A meta‐analysis. Global Change Biology, 22, 180–189. 10.1111/gcb.13028 26149723

[ece39460-bib-0055] Jonsson, B. , & Jonsson, N. (2001). Polymorphism and speciation in Arctic charr. Journal of Fish Biology, 58, 605–638.

[ece39460-bib-0056] Justine, J. L. , Briand, M. J. , & Bray, R. A. (2012). A quick and simple method, usable in the field, for collecting parasites in suitable condition for both morphological and molecular studies. Parasitology Research, 111, 341–351. 10.1007/s00436-012-2845-2846 22327319

[ece39460-bib-0057] Karvonen, A. , Bagge, A. , & Valtonen, E. (2005). Parasite assemblages of crucian carp (*Carassius carassius*)‐is depauperate composition explained by lack of parasite exchange, extreme environmental conditions or host unsuitability? Parasitology, 131, 273–278. 16145944 10.1017/s0031182005007572

[ece39460-bib-0058] Katoh, K. , Kuma, K. , Toh, H. , & Miyata, T. (2005). MAFFT version 5: Improvement in accuracy of multiple sequence alignment. Nucleic Acids Research, 33, 511–518. 10.1093/nar/gki198 15661851 PMC548345

[ece39460-bib-0059] Kearse, M. , Moir, R. , Wilson, A. , Stones‐Havas, S. , Cheung, M. , Sturrock, S. , Buxton, S. , Cooper, A. , Markowitz, S. , Duran, C. , Thierer, T. , Ashton, B. , Meintjes, P. , & Drummond, A. (2012). Geneious basic: An integrated and extendable desktop software platform for the organization and analysis of sequence data. Bioinformatics, 28, 1647–1649. 10.1093/bioinformatics/bts199 22543367 PMC3371832

[ece39460-bib-0060] Kelly, D. W. , Paterson, R. A. , Townsend, C. R. , Poulin, R. , & Tompkins, D. M. (2009). Parasite spillback: A neglected concept in invasion ecology? Ecology, 90, 2047–2056. 10.1890/08-1085.1 19739367

[ece39460-bib-0061] Kennedy, C. R. (1978). An analysis of the metazoan parasitocoenoses of brown trout *Salmo trutta* from British lakes. Journal of Fish Biology, 13, 255–263.

[ece39460-bib-0062] Kennedy, C. R. (2001). Metapopulation and community dynamics of helminth parasites of eels *Anguilla Anguilla* in the River Exe system. Parasitology, 122, 689–698. 11444622 10.1017/s0031182001007879

[ece39460-bib-0063] Kernan, M. , Battarbee, R. W. , & Moss, B. R. (2011). Climate change impacts on freshwater ecosystems. John Wiley & Sons.

[ece39460-bib-0064] Kleiber, C. , & Zeileis, A. (2008). Applied econometrics with R. Springer‐Verlag.

[ece39460-bib-0065] Klemetsen, A. , Amundsen, P. A. , Dempson, J. B. , Jonsson, B. , Jonsson, N. , O'Connell, M. F. , & Mortensen, E. (2003). Atlantic salmon *Salmo salar* L., brown trout *Salmo trutta* L. and Arctic charr *Salvelinus alpinus* (L.): A review of aspects of their life histories. Ecology of Freshwater Fish, 12, 1–59. 10.1034/j.1600-0633.2003.00010.x

[ece39460-bib-0066] Knudsen, R. , Amundsen, P. A. , Eloranta, A. P. , Hayden, B. , Siwertsson, A. , & Klemetsen, A. (2016). Parallel evolution of profundal Arctic charr morphs in two contrasting fish communities. Hydrobiologia, 783, 239–248. 10.1007/s10750-016-2647-4

[ece39460-bib-0067] Knudsen, R. , Amundsen, P. A. , & Klemetsen, A. (2003). Inter‐and intra‐morph patterns in helminth communities of sympatric whitefish morphs. Journal of Fish Biology, 62, 847–859.

[ece39460-bib-0068] Knudsen, R. , Amundsen, P.‐A. , Nilsen, R. , Kristoffersen, R. , & Klemetsen, A. (2007). Food borne parasites as indicators of trophic segregation between Arctic charr and brown trout. Environmental Biology of Fishes, 83, 107–116. 10.1007/s10641-007-9216-7

[ece39460-bib-0069] Knudsen, R. , Eloranta, A. P. , Siwertsson, A. , Paterson, R. A. , Power, M. , & Sandlund, O. T. (2019). Introduction of *Mysis relicta* (Mysida) reduces niche segregation between deep‐water Arctic charr morphs. Hydrobiologia, 840, 245–260. 10.1007/s10750-019-3953-4

[ece39460-bib-0070] Knudsen, R. , Gjelland, K. O. , Eloranta, A. P. , Hayden, B. , Siwertsson, A. , Amundsen, P. A. , & Klemetsen, A. (2016). A specialised cannibalistic Arctic charr morph in the piscivore guild of a subarctic lake. Hydrobiologia, 783, 65–78. 10.1007/s10750-015-2601-x

[ece39460-bib-0071] Knudsen, R. , Kristoffersen, R. , & Amundsen, P.‐A. (1997). Parasite communities in two sympatric morphs of Arctic charr, *Salvelinus alpinus* (L.), in northern Norway. Canadian Journal of Zoology, 75, 2003–2009.

[ece39460-bib-0072] Knudsen, R. , Siwertsson, A. , Adams, C. E. , Newton, J. , & Amundsen, P. A. (2014). Similar patterns of individual niche use are revealed by different time‐integrated trophic tracers (stable isotopes and parasites). Ecology of Freshwater Fish, 23, 259–268. 10.1111/eff.12074

[ece39460-bib-0073] Krebs, C. J. (1999). Ecological methodology. Benjamin/Cummings.

[ece39460-bib-0074] Kuchta, R. , Choudhury, A. , & Scholz, T. (2018). Asian fish tapeworm: The most successful invasive parasite in freshwaters. Trends in Parasitology, 34, 511–523. 10.1016/j.pt.2018.03.001 29580663

[ece39460-bib-0075] Kuhn, J. A. , Knudsen, R. , Kristoffersen, R. , Primicerio, R. , & Amundsen, P. A. (2016). Temporal changes and between–host variation in the intestinal parasite community of Arctic charr in a subarctic lake. Hydrobiologia, 783, 79–91. 10.1007/s10750-016-2731-9

[ece39460-bib-0076] Kuhn, J. A. , Kristoffersen, R. , Knudsen, R. , Jakobsen, J. , Marcogliese, D. J. , Locke, S. A. , Primicerio, R. , & Amundsen, P.‐A. (2015). Parasite communities of two three‐spined stickleback populations in subarctic Norway – effects of a small spatial‐scale host introduction. Parasitology Research, 114, 1327–1339. 25630694 10.1007/s00436-015-4309-2

[ece39460-bib-0077] L'Abée‐Lund, J. , Langeland, A. , & Sægrov, H. (1992). Piscivory by brown trout *Salmo trutta* L. and Arctic charr *Salvelinus alpinus* (L.) in Norwegian lakes. Journal of Fish Biology, 41, 91–101.

[ece39460-bib-0078] Langeland, A. , L'Abée‐Lund, J. , Jonsson, B. , & Jonsson, N. (1991). Resource partitioning and niche shift in Arctic charr *Salvelinus alpinus* and brown trout *Salmo trutta* . The Journal of Animal Ecology, 60, 895–912.

[ece39460-bib-0079] Lockyer, A. E. , Olson, P. D. , & Littlewood, D. T. J. (2003). Utility of complete large and small subunit rRNA genes in resolving the phylogeny of the Neodermata (Platyhelminthes): Implications and a review of the cercomer theory. Biological Journal of the Linnean Society, 78, 155–171. 10.1046/j.1095-8312.2003.00141.x

[ece39460-bib-0080] Lopez, L. K. , & Duffy, M. A. (2021). Mechanisms by which predators mediate host–parasite interactions in aquatic systems. Trends in Parasitology, 37, 890–906. 34281798 10.1016/j.pt.2021.06.006

[ece39460-bib-0081] Lysy, M. , Stasko, A. , & Swanson, H. (2014). nicheROVER: (Niche) (R)egion and Niche (Over)lap metrics for multidimensional ecological niches. R package version, 1 .

[ece39460-bib-0082] Mariotti, A. (1983). Atmospheric nitrogen is a reliable standard for natural 15N abundance measurements. Nature, 303, 685–687.

[ece39460-bib-0083] McCarthy, I. , Fraser, D. , Waldron, S. , & Adams, C. (2004). A stable isotope analysis of trophic polymorphism among Arctic charr from Loch Ericht, Scotland. Journal of Fish Biology, 65, 1435–1440.

[ece39460-bib-0084] Miller, M. A. , Pfeiffer, W. , & Schwartz, T. (2011). The CIPRES science gateway: A community resource for phylogenetic analyses. In Proceedings of the 2011 teragrid conference: Extreme digital discovery (pp. 1–8). ACM.

[ece39460-bib-0085] Moccetti, P. , Siwertsson, A. , Kjaer, R. , Amundsen, P. A. , Praebel, K. , Tamayo, A. M. P. , Power, M. , & Knudsen, R. (2019). Contrasting patterns in trophic niche evolution of polymorphic Arctic charr populations in two subarctic Norwegian lakes. Hydrobiologia, 840, 281–299. 10.1007/s10750-019-3969-9

[ece39460-bib-0086] Moravec, F. (2001a). Common sculpin *Cottus gobio* as a natural paratenic host of *Proteocephalus longicollis* (Cestoda: Proteocephalidae), a parasite of salmonids, in Europe. Diseases of Aquatic Organisms, 45, 155–158. 10.3354/dao045155 11463104

[ece39460-bib-0087] Moravec, F. (2001b). Trichinelloid nematode parasitic in cold–blooded vertebrates. Academy of Sciences of the Czech Republic.

[ece39460-bib-0088] Moravec, F. (2004). Metazoan parasites of Samonid fishes of Europe. Academia.

[ece39460-bib-0089] Moszczynska, A. , Locke, S. A. , McLaughlin, J. D. , Marcogliese, D. J. , & Crease, T. J. (2009). Development of primers for the mitochondrial cytochrome c oxidase I gene in digenetic trematodes (Platyhelminthes) illustrates the challenge of barcoding parasitic helminths. Molecular Ecology Resources, 9, 75–82. 21564967 10.1111/j.1755-0998.2009.02634.x

[ece39460-bib-0090] Nagelkerken, I. , Goldenberg, S. U. , Ferreira, C. M. , Ullah, H. , & Connell, S. D. (2020). Trophic pyramids reorganize when food web architecture fails to adjust to ocean change. Science, 369, 829–832. 32792395 10.1126/science.aax0621

[ece39460-bib-0091] Oksanen, J. , Blanchet, F. , Friendly, M. , Kindt, R. , Legendre, P. , McGlinn, D. , Minchin, P. , O’Hara, R. , Simpson, G. , & Solymos, P. (2020). Vegan: community ecology package. R package version 2.5‐7. https://cran.r‐project.org/package=vegan

[ece39460-bib-0093] Paterson, R. A. , Knudsen, R. , Blasco‐Costa, I. , Dunn, A. M. , Hytterod, S. , & Hansen, H. (2018). Determinants of parasite distribution in Arctic charr populations: Catchment structure versus dispersal potential. Journal of Helminthology, 1–8, 559–566. 10.1017/s0022149x18000482 29911512

[ece39460-bib-0094] Peeler, E. J. , Oidtmann, B. C. , Midtlyng, P. J. , Miossec, L. , & Gozlan, R. E. (2011). Non‐native aquatic animals introductions have driven disease emergence in Europe. Biological Invasions, 13, 1291–1303.

[ece39460-bib-0095] Penttinen, O.‐P. , & Holopainen, I. J. (1992). Seasonal feeding activity and ontogenetic dietary shifts in crucian carp, Carassius carassius. In W. Wieser , F. Schiemer , A. Goldschmidt , & K. Kotrschal (Eds.), Environmental biology of European cyprinids. Developments in environmental biology of fishes (Vol. 13, pp. 215–222). Springer.

[ece39460-bib-0096] Petchey, O. L. , McPhearson, P. T. , Casey, T. M. , & Morin, P. J. (1999). Environmental warming alters food‐web structure and ecosystem function. Nature, 402, 69–72.

[ece39460-bib-0097] Poulin, R. , & Mouillot, D. (2003). Host introductions and the geography of parasite taxonomic diversity. Journal of Biogeography, 30, 837–845. 10.1046/j.1365-2699.2003.00868.x

[ece39460-bib-0098] Power, M. , O'Connell, M. F. , & Dempson, J. B. (2005). Ecological segregation within and among Arctic char morphotypesin Gander Lake, Newfoundland. Environmental Biology of Fishes, 73, 263–274.

[ece39460-bib-0099] Præbel, K. , Couton, M. , Knudsen, R. , & Amundsen, P. A. (2016). Genetic consequences of allopatric and sympatric divergence in Arctic charr (*Salvelinus alpinus* (L.)) from Fjellfrøsvatn as inferred by microsatellite markers. Hydrobiologia, 783, 257–267. 10.1007/s10750-016-2648-3

[ece39460-bib-0100] R Core Team . (2021). R: A language and environment for statistical computing. R Core Team.

[ece39460-bib-0101] Reid, A. J. , Carlson, A. K. , Creed, I. F. , Eliason, E. J. , Gell, P. A. , Johnson, P. T. , Kidd, K. A. , MacCormack, T. J. , Olden, J. D. , & Ormerod, S. J. (2019). Emerging threats and persistent conservation challenges for freshwater biodiversity. Biological Reviews, 94, 849–873. 30467930 10.1111/brv.12480

[ece39460-bib-0102] Revelle, W. (2011). An overview of the psych package (p. 80). CRAN.

[ece39460-bib-0103] Romo, S. (1990). Seasonal zooplankton patterns in a shallow oligotrophic lake: Loch Rusky (Scotland). Annales de Limnologie‐International Journal of Limnology, 26, 11–17.

[ece39460-bib-0104] Ronquist, F. , Teslenko, M. , van der Mark, P. , Ayres, D. L. , Darling, A. , Höhna, S. , Larget, B. , Liu, L. , Suchard, M. A. , & Huelsenbeck, J. P. (2012). MrBayes 3.2: Efficient bayesian phylogenetic inference and model choice across a large model space. Systematic Biology, 61, 539–542. 10.1093/sysbio/sys029 22357727 PMC3329765

[ece39460-bib-0105] Saksvik, M. , Nilsen, F. , Nylund, A. , & Berland, B. (2001). Effect of marine *Eubothrium* sp. (Cestoda: Pseudophyllidea) on the growth of Atlantic salmon, *Salmo salar* L. Journal of Fish Diseases, 24, 111–119.

[ece39460-bib-0106] Sandlund, O. T. , Eloranta, A. P. , Borgstrøm, R. , Hesthagen, T. , Johnsen, S. I. , Museth, J. , & Rognerud, S. (2016). The trophic niche of Arctic charr in large southern Scandinavian lakes is determined by fish community and lake morphometry. Hydrobiologia, 783, 117–130.

[ece39460-bib-0107] Sandlund, O. T. , Gunnarsson, K. , Jónasson, P. M. , Jonsson, B. , Lindem, T. , Magnússon, K. P. , Malmquist, H. J. , Sigurjónsdóttir, H. , Skúlason, S. , & Snorrason, S. S. (1992). The arctic charr *Salvelinus alpinus* in Thingvallavatn. Oikos, 64, 305–351.

[ece39460-bib-0108] Scholz, T. (1999). Life cycles of species of *Proteotephalus*, parasites of fishes in the Palearctic Region: A review. Journal of Helminthology, 73, 1–20. 10431368

[ece39460-bib-0109] Scholz, T. , & Hanzelova, V. (1998). Tapeworms of the genus Proteocephalus Weinland, 1858 (Cestoda: Proteocephalidae), parasites of fishes in Europe. Academia.

[ece39460-bib-0110] Simonsen, M. K. , Siwertsson, A. , Adams, C. E. , Amundsen, P. A. , Praebel, K. , & Knudsen, R. (2017). Allometric trajectories of body and head morphology in three sympatric Arctic charr (*Salvelinus alpinus* (L.)) morphs. Ecology and Evolution, 7, 7277–7289. 10.1002/ece3.3224 28944016 PMC5606865

[ece39460-bib-0111] Siwertsson, A. , Refsnes, B. , Frainer, A. , Amundsen, P. A. , & Knudsen, R. (2016). Divergence and parallelism of parasite infections in Arctic charr morphs from deep and shallow lake habitats. Hydrobiologia, 783, 131–143. 10.1007/s10750-015-2563-z

[ece39460-bib-0112] Skúlason, S. , Noakes, D. L. , & Snorrason, S. S. (1989). Ontogeny of trophic morphology in four sympatric morphs of Arctic charr *Salvelinus alpinus* in Thingvallavatn, Iceland. Biological Journal of the Linnean Society, 38, 281–301.

[ece39460-bib-0113] Soldánová, M. , Georgieva, S. , Rohacova, J. , Knudsen, R. , Kuhn, J. A. , Henriksen, E. H. , Siwertsson, A. , Shaw, J. C. , Kuris, A. M. , Amundsen, P. A. , Scholz, T. , Lafferty, K. D. , & Kostadinova, A. (2017). Molecular analyses reveal high species diversity of trematodes in a sub‐Arctic lake. International Journal for Parasitology, 47, 327–345. 10.1016/j.ijpara.2016.12.008 28315362

[ece39460-bib-0114] Stamatakis, A. (2006). RAxML–VI–HPC: Maximum likelihood‐based phylogenetic analyses with thousands of taxa and mixed models. Bioinformatics, 22, 2688–2690. 10.1093/bioinformatics/btl446 16928733

[ece39460-bib-0115] Svenning, M. A. , Falkegård, M. , Dempson, J. B. , Power, M. , Bårdsen, B. J. , Guðbergsson, G. , & Fauchald, P. (2022). Temporal changes in the relative abundance of anadromous Arctic charr, brown trout, and Atlantic salmon in northern Europe: Do they reflect changing climates? Freshwater Biology, 67, 64–77.

[ece39460-bib-0116] Swanson, H. K. , Lysy, M. , Power, M. , Stasko, A. D. , Johnson, J. D. , & Reist, J. D. (2015). A new probabilistic method for quantifying *n*‐dimensional ecological niches and niche overlap. Ecology, 96, 318–324. 26240852 10.1890/14-0235.1

[ece39460-bib-0117] Taylor, E. B. , Boughman, J. W. , Groenenboom, M. , Sniatynski, M. , Schluter, D. , & Gow, J. L. (2006). Speciation in reverse: Morphological and genetic evidence of the collapse of a three‐spined stickleback (*Gasterosteus aculeatus*) species pair. Molecular Ecology, 15, 343–355. 16448405 10.1111/j.1365-294X.2005.02794.x

[ece39460-bib-0118] Tummers, B. (2006). DataThief III . https://datathief.org

[ece39460-bib-0119] Venables, W. N. , & Ripley, B. D. (2002). Modern applied statistics with S (5th ed.). Springer.

[ece39460-bib-0120] Verspoor, E. , Knox, D. , Greer, R. , & Hammar, J. (2010). Mitochondrial DNA variation in Arctic charr (*Salvelinus alpinus* (L.)) morphs from Loch Rannoch, Scotland: Evidence for allopatric and peripatric divergence. Hydrobiologia, 650, 117–131.

[ece39460-bib-0121] Waeschenbach, A. , Brabec, J. , Scholz, T. , Littlewood, D. T. J. , & Kuchta, R. (2017). The catholic taste of broad tapeworms–multiple routes to human infection. International Journal for Parasitology, 47, 831–843. 28780153 10.1016/j.ijpara.2017.06.004

[ece39460-bib-0122] Walker, A. , Greer, R. , & Gardner, A. (1988). Two ecologically distinct forms of Arctic charr *Salvelinus alpinus* (L.) in Loch Rannoch, Scotland. Biological Conservation, 43, 43–61.

[ece39460-bib-0123] Wallace, R. K., Jr. (1981). An assessment of diet‐overlap indexes. Transactions of the American Fisheries Society, 110, 72–76.

[ece39460-bib-0124] Woodward, G. , Perkins, D. M. , & Brown, L. E. (2010). Climate change and freshwater ecosystems: Impacts across multiple levels of organization. Philosophical Transactions of the Royal Society B: Biological Sciences, 365, 2093–2106. 10.1098/rstb.2010.0055 PMC2880135 20513717

