# Supplementary material for: Temporal stability of polymorphic Arctic charr parasite communities reflects sustained divergent trophic niches
Source: Ecol Evol. 2022 Nov 5;12(11):e9460. doi: 10.1002/ece3.9460 (PMC9636502; doi:10.1002/ece3.9460)
Supplement: Supplementary file 1 — Table S1. Table S2. Table S3. [file ECE3-12-e9460-s001.doc]

# Supplementary

Table S1: Taxa included in the molecular phylogenetic analyses, their host, localities and GenBank accession numbers. Taxa in bold have been newly sequence for this study and taxa highlighted in grey have been use for rooting their respecting tree.

| **Species** | **Location** | **Host** | **GenBank ID** |
| --- | --- | --- | --- |
| **TREMATODA – Allocreadiidae** | | | |
| ***Crepidostomum pseudifarionis*** | **Scotland: Loch Rannoch** | ***Salvelinus alpinus*** | **OP580487** |
| *Crepidostomum pseudofarionis* | Iceland: Lake Hafravatn | *Salvelinus alpinus* | MT080789 |
| *Crepidostomum farionis* | Norway: Lake Takvatn | *Sphaerium* sp. | KY513136 |
| *Crepidostomum brinkmanni* | Iceland: Lake Hafravatn | *Salmo trutta* | MT080773 |
| *Crepidostomum metoecus* | Russia, River near Nikolaevsk- na-Amure city | *Salvelinus leucomaensis* | FR821405 |
| *Crepidostomum nemachilus* | Russia, River near Nikolaevsk- na-Amure city | *Barbatula toni* | FR821408 |
| *Crepidostomum oschmarini* | Russia: River Il'd, upper Volga River basin | *Cottus gobio* | MH159989 |
| *Crepidostomum auritum* | USA: Pearl River, Mississippi | *Aplodinotus grunniens* | KF356373 |
| *Crepidostomum cornutum* | USA | *Lepomis gulosus* | EF032695 |
| *Crepidostomum illinoiense* | USA: Red Lake River, Minnesota | *Hiodon alosoides* | KF356372 |
| *Crepidostomum affine* | USA: Pearl River, Mississippi | *Aplodinotus grunniens* | KF356363 |
| *Crepidostomum auriculatum* | Russia, River near Nikolaevsk- na-Amure city | *Huso dauricus* | FR821397 |
| *Allocreadium neotenicum* | Norway: Lake Takvatn | *Pisidium casertanum* | MH143104 |
| *Allocreadium gotoi* | Japan: Nagano, Iiyama, Midori | *Misgurnus anguillicaudatus* | LC215274 |
| **TREMATODA - Diplostomoidea** | | | |
| ***Diplostomum baeri*** | **Scotland: Loch Rannoch** | ***Salvelinus alpinus*** | **OP577853** |
| ***Diplostomum baeri*** | **Scotland: Loch Rannoch** | ***Salvelinus alpinus*** | **OP577854** |
| ***Diplostomum baeri*** | **Scotland: Loch Rannoch** | ***Salvelinus alpinus*** | **OP577857** |
| ***Diplostomum baeri*** | **Scotland: Loch Rannoch** | ***Salvelinus alpinus*** | **OP577858** |
| ***Diplostomum baeri*** | **Scotland: Loch Rannoch** | ***Salvelinus alpinus*** | **OP577859** |
| ***Diplostomum baeri*** | **Scotland: Loch Rannoch** | ***Salvelinus alpinus*** | **OP577861** |
| ***Diplostomum baeri*** | **Scotland: Loch Rannoch** | ***Salvelinus alpinus*** | **OP577862** |
| ***Diplostomum* sp.** | **Scotland: Loch Rannoch** | ***Salvelinus alpinus*** | **OP577855** |
| ***Diplostomum* sp.** | **Scotland: Loch Rannoch** | ***Salvelinus alpinus*** | **OP577856** |
| ***Diplostomum* sp.** | **Scotland: Loch Rannoch** | ***Salvelinus alpinus*** | **OP577860** |
| *Diplostomum baeri* | Germany: River Ruhr (Henne) | *Salmo trutta fario* | JX986868 |
| *Diplostomum baeri* | Germany: Lake Constance | *Perca fluviatilis* | JQ639186 |
| *Diplostomum* sp. 1 | Canada: Manitoba, Lake Manitoba, South shore, Delta Marsh | *Larus argentatus* | HM064678 |
| *Diplostomum* sp. 2 | Canada: Quebec, St. Lawrence River, Lake Saint-Pierre, Sorel-Tracy | *Notropis hudsonius* | HM064691 |
| *Diplostomum* sp. 3 | Canada: Ontario | *Larus delawarensisa* | FJ477199 |
| *Diplostomum* sp. 4 | Canada | *Larus delawarensis* | GQ292494 |
| *Diplostomum* sp. 5 | Canada | *Perca flavescens* | GQ292498 |
| *Diplostomum* sp. 6 | Canada | *Pimephales notatus* | GQ292499 |
| *Diplostomum* sp. 7 | Canada | *Pimephales notatus* | GQ292500 |
| *Diplostomum* sp. 8 | Canada | *Rana pipiens* | GQ292497 |
| *Diplostomum* sp. 9 | Canada | *Percina caprodes* | GQ292496 |
| *Diplostomum spathaceum* | Germany: Hengsteysee | *Radix auricularia* | JX986891 |
| *Diplostomum spathaceum* | Czech Republic: near Tovacov | *Larus cachinnans* | JX986895 |
| *Diplostomum pseudospathaceum* | Germany: Hengsteysee | *Gasterosteus aculeatus* | JX986903 |
| *Diplostomum mergi* | Germany: Hengsteysee | *Radix auricularia* | JX986873 |
| *Diplostomum mergi* | Germany: River Ruhr (Henne) | *Salmo trutta fario* | JX986881 |
| *Diplostomum indistinctum* | Canada: Quebec | *Catostomus commersoni* | FJ477196 |
| *Diplostomum indistinctum* | Canada: Quebec, St. Lawrence River, Lake Saint Louis, Dorval Island | *Catostomus commersoni* | HM064673 |
| *Diplostomum huronense* | Canada: Ontario, Lake Ontario, Near Kingston | *Larus argentatus* | HM064667 |
|  |  |  |  |
| *Tylodelphys clavatas* | Germany: Hengsteysee | *Radix auricularia* | JX986908 |
| *Tylodelphys clavatas* | Germany: River Lippe | *Perca fluviatilis* | JX986909 |
| **ACANTHOCEPHALA - *Echinorhynchidae*** | | | |
| ***Echinorhynchus truttae*** | **Scotland: Loch Rannoch** | ***Salvelinus alpinus*** | **OP580482** |
| ***Echinorhynchus truttae*** | **Scotland: Loch Rannoch** | ***Salvelinus alpinus*** | **OP580483** |
| ***Echinorhynchus truttae*** | **Scotland: Loch Rannoch** | ***Salvelinus alpinus*** | **OP580484** |
| ***Echinorhynchus truttae*** | **Scotland: Loch Rannoch** | ***Salvelinus alpinus*** | **OP580485** |
| ***Echinorhynchus truttae*** | **Scotland: Loch Rannoch** | ***Salvelinus alpinus*** | **OP580486** |
| *Echinorhynchus truttae* | UK: Loch Walton Burn, River Carron catchment, Scotland | *Salmo trutta* | KM656147 |
| *Echinorhynchus bothniensis* | Finland: Lake Keitele | *Osmerus eperlanus* | KM656146 |
| *Echinorhynchus gadi* | Russia: White Sea, Gulf of Kandalaksha, Chupa Inlet | *Gadus morhua* | KM656150 |
| *Echinorhynchus brayi* | Atlantic Ocean: Porcupine Seabight | *Pachycara crassiceps* | KM656151 |
| *Echinorhynchus* sp. | Switzerland: Buochs, Scheidgraben stream | *Salmo trutta* | MT738710 |
| *Echinorhynchus cinctulus* | Finland: Kuopio | *Lota lota* | KM656142 |
| *Echinorhynchus salmonis* | Finland: Baltic Sea, Bothnian Bay | *Coregonus lavaretus* | KM656145 |
| *Pomphorhynchus bulbocolli* | - | *Lepomis macrochirus* | AY829096 |
| *Acanthocephalus lucii* | UK: Bleasby, Nottinghamshire | *Perca fluviatilis* | KM656148 |
| *Acanthocephalus lucii* | UK: Bleasby, Nottinghamshire | *Perca fluviatilis* | KM656148 |

Table S2. Influence of morph and fish length (measured to the fork, mm) on the total parasite abundance (number of individual parasites) and taxa richness infecting Arctic charr in Loch Rannoch, Scotland, UK.

| Index | Morph | Variable level | Estimate | SE | *t* value | *P* |
| --- | --- | --- | --- | --- | --- | --- |
| **Total abundance^** | BE | Intercept | -1.60 | 1.27 | -1.26 | 0.210 |
|  |  | PI | 1.17 | 1.50 | 0.78 | 0.436 |
|  |  | PL | 1.24 | 2.20 | 0.56 | 0.574 |
|  |  | **Length** | **0.01** | **0.01** | **2.71** | **0.008** |
|  |  | PI x Length | -0.00 | 0.01 | -0.30 | 0.769 |
|  |  | PL x Length | -0.00 | 0.01 | -0.11 | 0.915 |
|  | PI | Intercept | -0.43 | 0.80 | -0.53 | 0.595 |
|  |  | BE | -1.17 | 1.50 | -0.78 | 0.436 |
|  |  | PL | 0.07 | 1,97 | 0.04 | 0.972 |
|  |  | **Length** | **0.01** | **0.00** | **4.81** | **6.44e-06** |
|  |  | BE x Length | 0.00 | 0.01 | 0.30 | 0.769 |
|  |  | PL x Length | 0.00 | 0.01 | 0.09 | 0.931 |
| **Taxa richness *S*** | BE | Intercept | 0.13 | 0.63 | 0.21 | 0.833 |
|  |  | PI | -0.16 | 0.90 | -0.18 | 0.856 |
|  |  | PL | -0.48 | 1.35 | -0.36 | 0.720 |
|  |  | Length | 0.00 | 0.00 | 0.87 | 0.385 |
|  |  | PI x Length | 0.00 | 0.00 | 0.41 | 0.680 |
|  |  | PL x Length | 0.00 | 0.01 | 0.40 | 0.683 |
|  | PI | Intercept | -0.03 | 0.64 | -0.05 | 0.962 |
|  |  | BE | 0.16 | 0.90 | 0.18 | 0.856 |
|  |  | PL | -0.32 | 1.36 | -0.24 | 0.813 |
|  |  | Length | 0.00 | 0.00 | 1.86 | 0.063 |
|  |  | BE x Length | -0.00 | 0.00 | 0.41 | 0.680 |
|  |  | PL x Length | 0.00 | 0.01 | 0.17 | 0.865 |

Note: BE Benthivore, PI Piscivore, PL Planktivore, ^model fitted with quasipoisson distribution, all other models fitted with Poisson. Length centred on the mean and scaled by one standard deviation.

Table S3. Between lake differences in abundance of major parasites taxa infecting Arctic charr in Loch Rannoch, Scotland, UK.

| Parasites taxa | Morph | Variable level | Estimate | SE | *t* value | *P* |
| --- | --- | --- | --- | --- | --- | --- |
| **Digenea** |  |  |  |  |  |  |
| *Crepidostomum* sp. | BE | Intercept | -1.64e+00 | 5.22e+00 | -0.31 | 0.755 |
| N=91 |  | PI | 1.08e+00 | 5.45e+00 | 0.20 | 0.844 |
|  |  | PL | 2.06e+02 | 2.72e+04 | 0.01 | 0.994 |
|  |  | Length | 2.78e-03 | 2.34e-02 | 0.12 | 0.906 |
|  |  | PI x Length | 5.97e-03 | 2.39e-02 | 0.25 | 0.804 |
|  |  | PL x Length | -1.50e+00 | 1.99e+02 | -0.01 | 0.994 |
|  | PI | Intercept | -5.60e-01 | 1.57e-00 | -0.36 | 0.722 |
|  |  | BE | -1.08e+00 | 5.45e+00 | -0.20 | 0.844 |
|  |  | PL | 2.05e+02 | 2.72e+04 | 0.01 | 0.994 |
|  |  | Length | 8.75e-03 | 5.00e-03 | 1.75 | 0.084 |
|  |  | Length x BE | -5.97e-03 | 2.39e-02 | -0.25 | 0.804 |
|  |  | Length x PL | -1.50e+00 | 1.99e+02 | -0.01 | 0.994 |
|  |  |  |  |  |  |  |
| *Diplostomum* spp.^ | BE | Intercept | -4.07 | 2.14 | -1.90 | 0,061 |
| *Tylodelphys* sp. |  | PI | 1.83 | 2.83 | 0.65 | 0.519 |
| N=101 |  | PL | -4.67 | 36.74 | -0.13 | 0.899 |
|  |  | **Length** | **0.02** | **0.01** | **2.60** | **0.011** |
|  |  | PI x Length | -0.01 | 0.01 | -0.76 | 0.447 |
|  |  | PL x Length | 0.00 | 0.15 | 0.03 | 0.978 |
|  | PI | Intercept | -2.24 | 1.85 | -1.21 | 0.231 |
|  |  | BE | -1.83 | 2.83 | -0.65 | 0.519 |
|  |  | PL | -6.50 | 36.72 | -0.18 | 0.860 |
|  |  | **Length** | **0.01** | **0.01** | **2.34** | **0.021** |
|  |  | Length x BE | 0.01 | 0.01 | 0.76 | 0.447 |
|  |  | Length x PL | 0.01 | 0.15 | 0.08 | 0.939 |
|  |  |  |  |  |  |  |
| **Cestoda** |  |  |  |  |  |  |
| *Proteocephalus longicolis**$ | BE | **Intercept** | **-3.87** | **1.37** | **-2.84** | **0.005** |
| N=58 |  | **PL** | **3.29** | **0.72** | **4.56** | **5.18e-06** |
|  |  | Length | 0.01 | 0.01 | 0.99 | 0.321 |
|  |  |  |  |  |  |  |
| *Eubothrium salvelini* | BE | **Intercept** | **-2.54** | **1.16** | **-2.19** | **0,029** |
| N=91 |  | PI | 0.85 | 1.67 | 0.51 | 0.612 |
|  |  | PL | 0.76 | 2.19 | 0.35 | 0.728 |
|  |  | Length | 0.01 | 0.01 | 1.79 | 0.074 |
|  |  | PI x Length | -0.00 | 0.01 | -0.52 | 0.601 |
|  |  | PL x Length | -0.00 | 0.01 | -0.08 | 0.937 |
|  | PI | Intercept | -1.69 | 1.20 | -1.41 | 0.158 |
|  |  | BE | -0.85 | 1.67 | -0.51 | 0.612 |
|  |  | PL | -0.09 | 2.21 | -0.04 | 0.969 |
|  |  | Length | 0.01 | 0.00 | 1.40 | 0.160 |
|  |  | Length x BE | 0.00 | 0.01 | 0.52 | 0.601 |
|  |  | Length x PL | 0.00 | 0.01 | 0.29 | 0.773 |
|  |  |  |  |  |  |  |
| *Dibothriocephalus* spp.^ | BE | Intercept | -1.83 | 2.64 | -0.69 | 0.491 |
| N=91 |  | PI | -0.81 | 2.83 | -0.29 | 0.775 |
|  |  | PL | 0.22 | 3.10 | 0.07 | 0.944 |
|  |  | Length | 0.01 | 0.01 | 0.46 | 0.644 |
|  |  | PI x Length | 0.01 | 0.01 | 0.88 | 0.382 |
|  |  | PL x Length | 0.01 | 0.01 | 0.86 | 0.390 |
|  | PI | **Intercept** | **-2.64** | **1.03** | **-2.57** | **0.012** |
|  |  | BE | 0.81 | 2.83 | 0.29 | 0.775 |
|  |  | PL | 1.03 | 1.93 | 0.54 | 0.593 |
|  |  | **Length** | **0.02** | **0.00** | **5.01** | **2.88e-06** |
|  |  | Length x BE | -0.01 | 0.01 | -0.88 | 0.382 |
|  |  | Length x PL | 0.00 | 0.01 | 0.14 | 0.888 |
| **Acanthocephala** |  |  |  |  |  |  |
| Acanthocephalans**$ | PI | Intercept | -1.91 | 1.95 | -0.98 | 0.327 |
| N=60 |  | PL | -1.94 | 1.14 | -1.71 | 0.087 |
|  |  | Length | 0.00 | 0.01 | 0.38 | 0.708 |
|  |  |  |  |  |  |  |
| **Nematoda** |  |  |  |  |  |  |
| *Pseudocapillaria* *salvelini*$ | BE | **Intercept** | **-1.96** | **0.95** | **-2.06** | **0.040** |
| N=91 |  | PI | -1.01 | 0.52 | -1.94 | 0.052 |
|  |  | **PL** | **-1.61** | **0.63** | **-2.55** | **0.011** |
|  |  | Length | 0.01 | 0.00 | 1.45 | 0.145 |
|  | PL | Intercept | **-2.97** | **1.29** | **-2.31** | **0.021** |
|  |  | BE | 1.01 | 0.52 | 1.94 | 0.052 |
|  |  | PL | -0.60 | 0.72 | -0.83 | 0.408 |
|  |  | Length | 0.01 | 0.00 | 1.45 | 0.146 |
|  |  |  |  |  |  |  |
| Unknown nematode*** | BE | Intercept | 0.64 | 2.24 | 0.28 | 0.776 |
| N=64 |  | PI | 4.13 | 5.33 | 0.78 | 0.438 |
|  |  | Length | -0.01 | 0.01 | -0.93 | 0.353 |
|  |  | Length x PI | -0.02 | 0.03 | -0.90 | 0.369 |
|  |  |  |  |  |  |  |
|  |  |  |  |  |  |  |

Note: BE Benthivore, PI Piscivore, PL Planktivore, *Parasite absent from Piscivore, **Parasite absent from Benthivore, ***Parasite absent from Planktivore, ^model fitted with QuasiPoisson distribution, all other models fitted with Poisson distribution. Length centred on the mean and scaled by one standard deviation. $ GLM model run without interaction between variables.
